# Proteome Analysis of Subsarcolemmal Cardiomyocyte Mitochondria: A Comparison of Different Analytical Platforms

**DOI:** 10.3390/ijms15069285

**Published:** 2014-05-26

**Authors:** Francesco Giorgianni, Diwa Koirala, Karl T. Weber, Sarka Beranova-Giorgianni

**Affiliations:** 1 Department of Pharmaceutical Sciences, the University of Tennessee Health Science Center, Memphis, TN 38163, USA; E-Mails: fgiorgia@uthsc.edu (F.G.); dkoirala@uthsc.edu (D.K.); 2 Division of Cardiology, Department of Medicine, the University of Tennessee Health Science Center, Memphis, TN 38163, USA; E-Mail: ktweber@uthsc.edu

**Keywords:** mitochondria, proteome, rat, cardiomyocyte, bioanalytical platform, protein identification, IEF, SDS-PAGE, PLRP, LC-MS/MS

## Abstract

Mitochondria are complex organelles that play critical roles in diverse aspects of cellular function. Heart disease and a number of other pathologies are associated with perturbations in the molecular machinery of the mitochondria. Therefore, comprehensive, unbiased examination of the mitochondrial proteome represents a powerful approach toward system-level insights into disease mechanisms. A crucial aspect in proteomics studies is design of bioanalytical strategies that maximize coverage of the complex repertoire of mitochondrial proteins. In this study, we evaluated the performance of gel-based and gel-free multidimensional platforms for profiling of the proteome in subsarcolemmal mitochondria harvested from rat heart. We compared three different multidimensional proteome fractionation platforms: polymeric reversed-phase liquid chromatography at high pH (PLRP), sodium dodecyl sulfate-polyacrylamide gel electrophoresis (SDS-PAGE), and isoelectric focusing (IEF) separations combined with liquid chromatography-mass spectrometry/mass spectrometry (LC-MS/MS), and bioinformatics for protein identification. Across all three platforms, a total of 1043 proteins were identified. Among the three bioanalytical strategies, SDS-PAGE followed by LC-MS/MS provided the best coverage of the mitochondrial proteome. With this platform, 890 proteins with diverse physicochemical characteristics were identified; the mitochondrial protein panel encompassed proteins with various functional roles including bioenergetics, protein import, and mitochondrial fusion. Taken together, results of this study provide a large-scale view of the proteome in subsarcolemmal mitochondria from the rat heart, and aid in the selection of optimal bioanalytical platforms for differential protein expression profiling of mitochondria in health and disease.

## 1. Introduction

Mitochondria are multifunctional organelles that play critical functional roles in eukaryotic cells, and impaired mitochondrial function has been implicated in a variety of diseases [1]. Mitochondrial dysfunction is associated with the pathophysiologic scenario leading to the failing heart phenotype [2,3]. Cardiomyocytes have a large percentage of mitochondria, constituting 30%–40% of cellular volume [4]. In the cardiomyocyte, distinct subpopulations of mitochondria exist: the subsarcolemmal mitochondria (SSM) are located immediately beneath the sarcolemma, and interfibrillar mitochondria (IFM) are situated between the myofibrils. These two subpopulations exhibit differences in their spatial localization, morphology, and biochemical properties [5]. The primary purpose of mitochondria is to provide bioenergy in the form of adenosine triphosphate (ATP) to fuel the pumping activity of the heart. Alterations in mitochondrial bioenergetics, including disturbance in substrate utilization and aberrations in oxidative phosphorylation, result in chronic energy deficit that contributes to compromised function of the failing heart [5,6]. The portfolio of important mitochondrial activities includes generation of reactive oxygen species (ROS) critical for intracellular signaling, which can also inflict self-injury when persistent and excessive [5].

Because of the multifaceted, highly interconnected nature of mitochondrial perturbations, there is a critical need to develop an integrated mechanistic understanding of cardiomyocyte mitochondrial dysfunction, as it relates to heart failure. In the absence of such comprehensive, systems-level information, our mechanistic knowledge will be fragmented and, consequently, design of improved treatment strategies will remain challenging. It is therefore fundamental to be able to interrogate the cardiomyocyte mitochondrial proteome at the deepest level possible.

Currently, no single analytical platform exists that is capable of obtaining the complete proteome map of an entire tissue, cell or even an organelle of a eukaryotic cell. Instead, multiple analytical approaches are required. Multi-dimensional chromatography and/or electrophoretic techniques are the most widely used analytical methods to pre-fractionate highly complex proteomes, thus reducing their complexity before liquid chromatography-mass spectrometry/mass spectrometry (LC-MS/MS) analysis. An almost infinite combination of upstream fractionation methods is possible, and selection of the analytical platform depends on the desired outcome, the number of samples to be analyzed, and resources and time available to complete the analyses [7]. In general, configurations used to achieve in-depth interrogation of complex systems include: fractionation of intact proteins and direct characterization by high-resolution mass spectrometry; fractionation of intact proteins in the first dimension followed by proteolytic digestions of the fractionated samples, and on-line reversed-phase (RP) LC-MS/MS analysis; or digestion of the sample followed by peptide fractionation in the first dimension followed by RP LC-MS/MS analysis of the fractionated samples. The first method, *i.e.*, fractionation of whole proteins and subsequent MS characterization without proteolytic digestion, the so called top-down approach, offers various advantages over methods that use proteolytic digestion prior to MS identification: (1) It allows for the identification of novel protein modifications and isoforms; (2) It provides valuable stoichiometric information on the ratio of the various isomers of the identified proteins [8]. Top-down methods, however, require high-resolution mass spectrometers and a relatively large amount of analytes. At the other extreme of the analytical spectrum of popular methods, there is the bottom-up proteomics approach where samples are first digested before various fractionation steps at the peptide level are performed. In this arena, multidimensional protein identification technology (MudPIT) is a popular 2D-LC analytical platform capable of identifying hundreds to thousands of protein species in complex sample matrices [9]. The MudPIT platform uses ion-exchange in the first dimension followed by on-line reversed-phase LC-MS/MS. The main advantages of this method include its purported orthogonality, *i.e.*, ion-exchange followed by C_18_reversed-phase (C_18_ RP), and ease of automation for high throughput LC-MS/MS analyses. An alternative method to MudPIT has emerged in recent years, namely the reversed-phase reversed-phase (RP–RP) platform for 2D LC separation of peptides. In the RP–RP method, a polymer-based reversed-phase (PLRP) column and high-pH mobile phase is used in the first dimension, followed by conventional C18 reversed-phase separation at low pH. A side-by-side comparative analysis of the MudPIT and RP–RP methods showed a superior performance by the RP–RP platform, due at least in part to the ability of the RP–RP method to separate very hydrophobic peptides, and to a better orthogonality between first and second dimensions [10]. The main drawback of the RP–RP method is represented by the inherent difficulty related to on-line automation. Despite this limitation, various studies have demonstrated the better performance of PLRP-based approaches in 2D LC-MS/MS applications for shotgun proteomic studies compared to the MudPIT platform [11,12]. Thus, the PLRP-based method represents a valid alternative to ion-exchange peptide separation in the first dimension of 2D LC-MS/MS analytical platforms. Although any peptide 2D LC method is effective at expanding the repertoire of a complex proteome, as compared to a single dimension LC-MS/MS, and because first and second dimensions are performed at the peptide level, various drawbacks are encountered, among them the loss of information on physicochemical features of the intact protein such as protein molecular weight and p*I*. A simple and effective approach to reduce the complexity of large proteomes that does not require high-end mass spectrometry resources and is capable of surveying thousands of proteins in a single sample, while preserving the link between identified peptides and intact proteins (*i.e.*, p*I* and molecular weight), is 2D gel electrophoresis (2DE). The major drawback of this strategy is the significant amount of labor required for gel fractionation, protein extraction, protein digestion and peptide purification. Furthermore, sensitivity of 2DE is limited by stain performance.

In recent years, to obtain in-depth characterization of complex proteomics samples while preserving the link between peptides and intact proteins features, we have used a strategy that relies on the simplicity and separation power of in-gel isoelectric focusing (IEF) for upstream protein fractionation [13,14,15]. In addition to the IEF method, SDS-PAGE has also been extensively used as an effective fractionation method to reduce the complexity of large proteomes for LC-MS/MS analysis. (When coupled to LC-MS/MS, this method is frequently referred to as GeLC-MS/MS [16]). These two electrophoretic methods, IEF and SDS-PAGE, represent powerful platforms for the fractionation of intact proteins before proteolytic digestion. The introduction of equipment like the OFF-Gel system (Agilent, Santa Clara, CA, USA), designed to automate the IEF-based protein separation method, have increased the popularity of these techniques among proteomics research laboratories across the globe [17].

In the study reported here, three different multidimensional bioanalytical platforms were used to analyze the proteome in subsarcolemmal mitochondria (SSM) isolated from rat cardiomyocytes. The main objective of the study was to evaluate the capabilities of these platforms in terms of extent and depth of proteome coverage. The three platforms incorporated different first-dimension separation methods—PLRP, in-gel IEF and SDS-PAGE—in combination with down-stream RP LC-MS/MS. Our investigation showed the superior performance of the SDS-PAGE-based platform. Survey of the SSM proteome with this platform provided access to the highest number of mitochondrial proteins over a wide range of abundance levels. The SSM proteome profile that was obtained encompasses proteins with diverse functional characteristics. Thus, among the configurations evaluated, the SDS-PAGE-based platform is indicated as the best choice as a multidimensional bioanalytical strategy for qualitative and quantitative mitochondrial proteome profiling.

## 2. Results

We conducted a systematic, comparative evaluation of three multidimensional analytical platforms for the characterization of the proteome in subsarcolemmal mitochondria isolated from rat cardiomyocytes. Prior to proteome analyses, the purity of the rat subsarcolemmal mitochondria preparations was assessed by flow cytometry and mitochondria-specific dye, as previously described [18].

**Figure 1:**
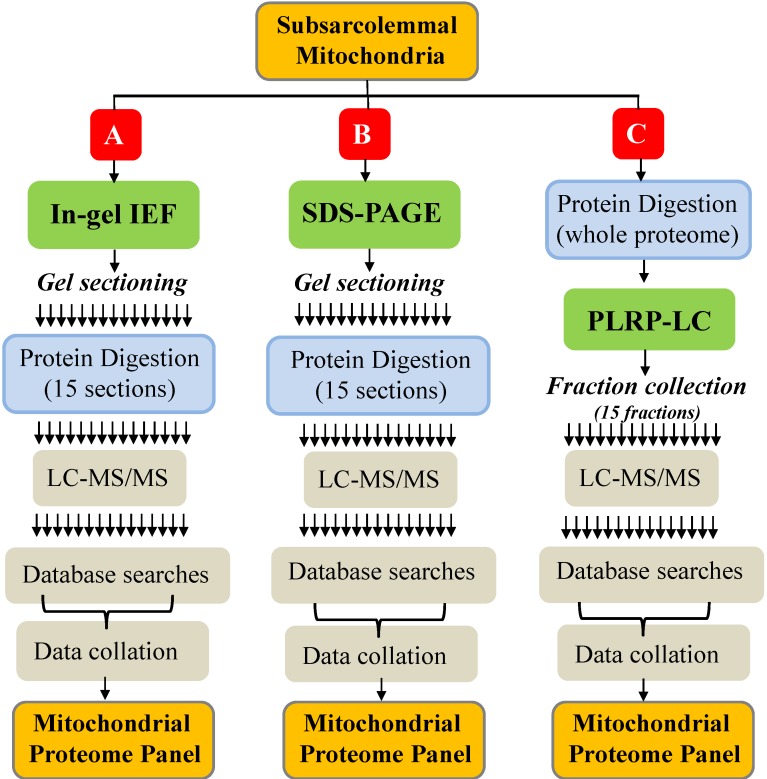
Experimental scheme for the comparative evaluation of three bioanalytical platforms for mitochondrial proteome profiling. Mitochondrial protein mixtures were separated at the protein level by in-gel isoelectric focusing (IEF) (**A**) or sodium dodecyl sulfate-polyacrylamide gel electrophoresis (SDS-PAGE) (**B**); or by PLRP (polymeric reversed-phase liquid chromatography at high pH) at the peptide level following proteolytic digestion (**C**). Fifteen gel sections (**A**,**B**) or 15 liquid fractions (**C**) were produced, and the peptide mixtures were analyzed by liquid chromatography-mass spectrometry/mass spectrometry (LC-MS/MS). The LC-MS/MS data were used to identify the proteins sampled with each platform. Three technical replicates were performed with each platform. Multiple arrows indicate multiple fractions/sections.

The overall experimental scheme is shown in Figure 1. The bioanalytical platforms evaluated in our study incorporated three different gel-based or gel-free pre-fractionation methods. The gel-based pre-fractionations were performed at the protein level using in-gel IEF or SDS-PAGE (Figure 1). Following protein separation, each gel was divided into 15 sections. The proteins in each section were digested with trypsin, and the tryptic peptide mixtures were analyzed by LC-MS/MS. The LC-MS/MS datasets were used in database searches to identify the proteins in each section, and the resulting protein identification lists were combined to yield the final mitochondrial protein catalog for each platform. For the gel-free separation, mitochondrial proteins were first digested with trypsin, and the separation was performed at the peptide level using reversed-phase LC at high pH (PLRP). Fifteen peptide fractions were collected and subjected to LC-MS/MS, database searches and data processing as described above. Technical replicates (*n* = 3) were performed with each gel-based and gel-free platform.

### 2.1. Summary of Protein Identification Results

As outlined in Figure 1, with each platform 15 peptide mixtures per replicate were analyzed by LC-MS/MS. In total, 135 LC-MS/MS analyses (runs) were performed in the study. The protein identification results are summarized in Table 1. Protein false discovery rates were calculated by Percolator using the rat reverse decoy database [19,20]. Positive peptide matches were assigned for fully tryptic peptides with PEP scores <0.01. Using the Swiss-Prot rat database, a total of 1043 unique, non-redundant mitochondrial proteins were identified across all platforms. Full lists of proteins identified with each platform are provided in Tables S1–S3. Previous global mitochondrial proteomics studies in rats on different tissues (skeletal muscle, heart, and liver) have characterized a total of 689 proteins [21].

**Figure 2:**
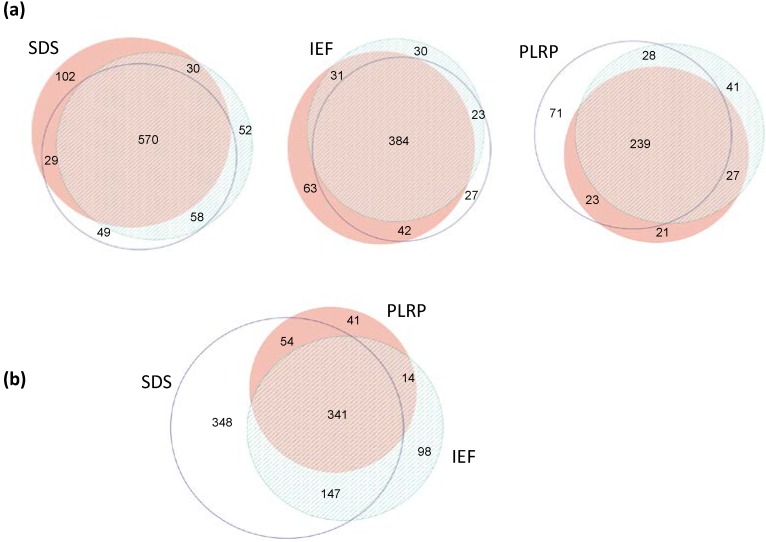
Venn diagrams showing distribution of unique proteins identified by each platform. (**a**) Top portion of the figure shows the overlap for each platform among the three technical replicates; (**b**) Bottom portion of the figure shows the overlap between the three individual platforms.

The performance of the different analytical platforms for mapping of the SSM proteome varied considerably. As shown in Table 1 and Figure 2, the overall proteome coverage overlap across all three platforms was 32.7%, with 341 proteins mapped across all methods. The SDS-PAGE-based platform yielded the highest number of unique identified proteins (890), the highest number of unique peptides (*i.e.*, highest protein coverage), the best precision and the highest proteome coverage overlap (together with the IEF) among technical replicates (Table 1 and Figure 2). The IEF-based platform yielded a total of 600 identified proteins; however, the total peptide count was only 50% compared with the SDS-PAGE (Table 1), indicating a decrease in protein coverage. The performance of the PLRP-based platform (separation at the peptide level) was, in our setting, decidedly below the SDS-PAGE and IEF-based platforms (protein-level separations).

**Table 1:** Summary of the performance of three gel-based and gel-free bioanalytical platforms for mitochondrial proteome mapping.

Performance Characteristic	SDS-PAGE	IEF	PLRP
Number of proteins—replicate 1	708	520	315
Number of proteins—replicate 2	726	468	330
Number of proteins—replicate 3	696	476	361
Average ± STD	710 ± 15	488 ± 28	335 ± 23
C.V.	2.1%	5.7%	7.0%
Total number of proteins (all runs)	890	600	450
Total number of peptides (all runs)	6023	3110	1671
Proteome coverage overlap	64%	63%	53%
Total number of proteins (all platforms)	1043	–	–
Proteome coverage overlap (all platforms)	32.7%	–	–

Abbreviations: SDS-PAGE, sodium dodecyl sulfate-polyacrylamide gel electrophoresis; IEF, isoelectric focusing; PLRP, polymeric reversed-phase liquid chromatography at high pH; C.V., coefficient of variation.

### 2.2. Intrinsic Chemical Properties of the Identified Proteins

**Figure 3:**
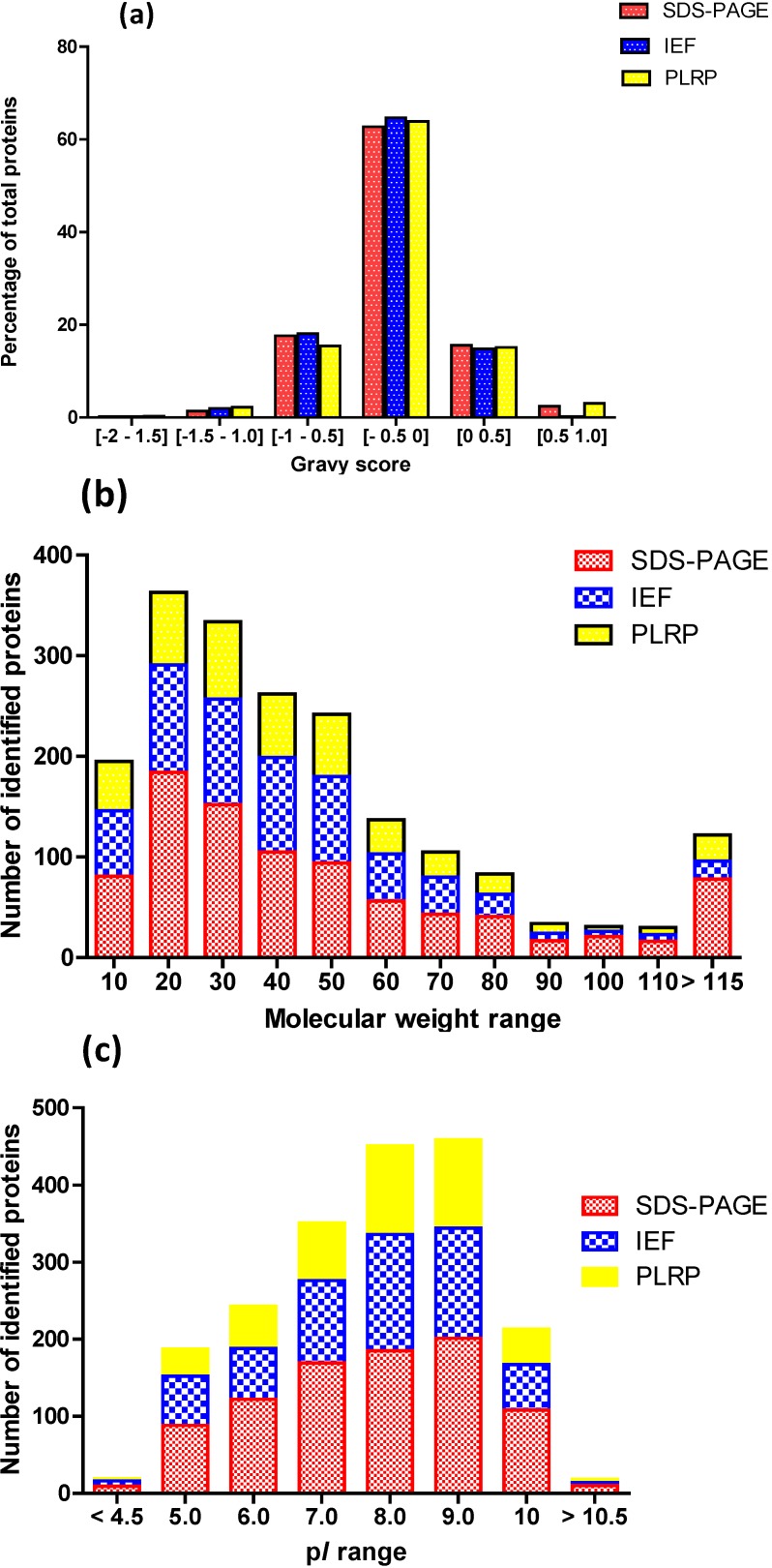
Distribution of intrinsic properties of the mitochondrial proteins identified with each platform. (**a**) grand average of hydropathy (GRAVY) score; (**b**) *M*_W_ (in kDa); (**c**) p*I*.

We analyzed the intrinsic chemical properties of the identified proteins: grand average of hydropathy (GRAVY) score, molecular weight (*M*_W_) and p*I* (Figure 3). Overall, these protein properties displayed similar trends among the three platforms. The only molecular feature that showed a significant difference between the three platforms relates to the high-*M*w section of the distribution (Figure 3b). In this category, the SDS-PAGE-based platform method allowed the identification of a statistically significant (*p* < 0.001) greater number of high-*M*_W_ proteins (12.9% ≥ 100 kDa). A second noteworthy observation regarding the intrinsic chemical properties, which is platform-independent, is the predominance of basic proteins (p*I* values between 7 and 9) in our mitochondrial proteome panel (Figure 3c).

### 2.3. Mitochondrial Proteome Abundance

**Figure 4:**
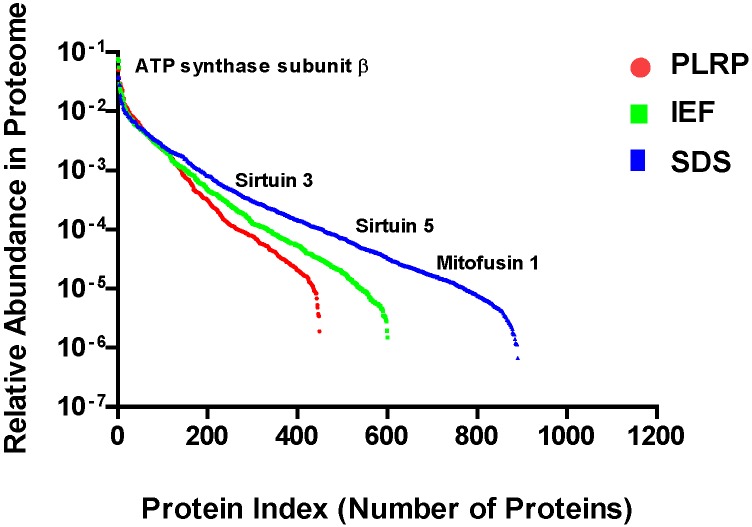
Relative abundance of mitochondrial proteins sampled with each platform.

To provide information about the dynamic range of protein coverage afforded by each platform, relative protein abundance was assessed using normalized spectral abundance factors (NSAF) [22]. As shown in Figure 4, all three platforms sampled proteins over a wide range of abundance levels. The SDS-PAGE-based platform provided the most in-depth survey of mitochondrial proteins with a dynamic range spanning five orders of magnitude (10^−1^–10^−6^), from the most abundant protein in the proteome, ATP synthase subunit β (*ca.* 3% of total protein), to medium-and low-abundance proteins such as sirtuins 3 and 5, and mitofusin 1.

### 2.4. Categorization of the Identified Proteins

**Figure 5:**
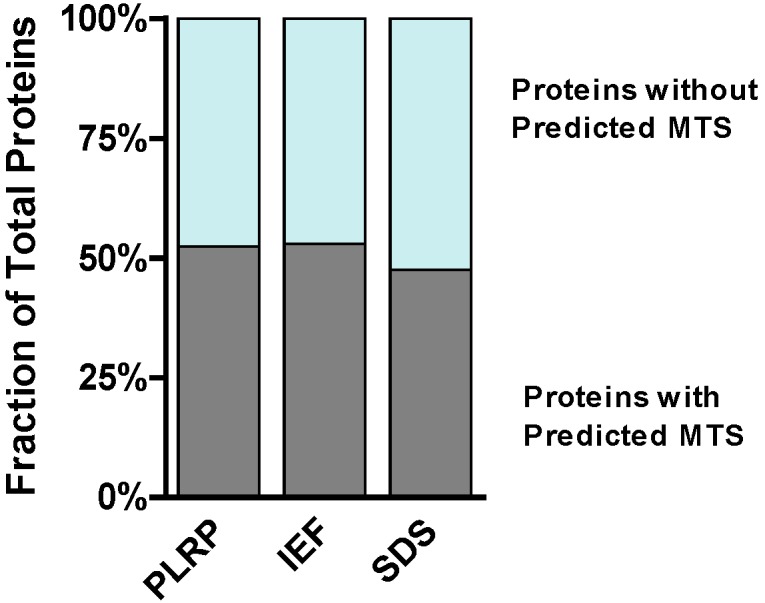
Distribution of proteins according to the presence of *N*-terminal mitochondrial targeting sequence (MTS).

**Figure 6:**
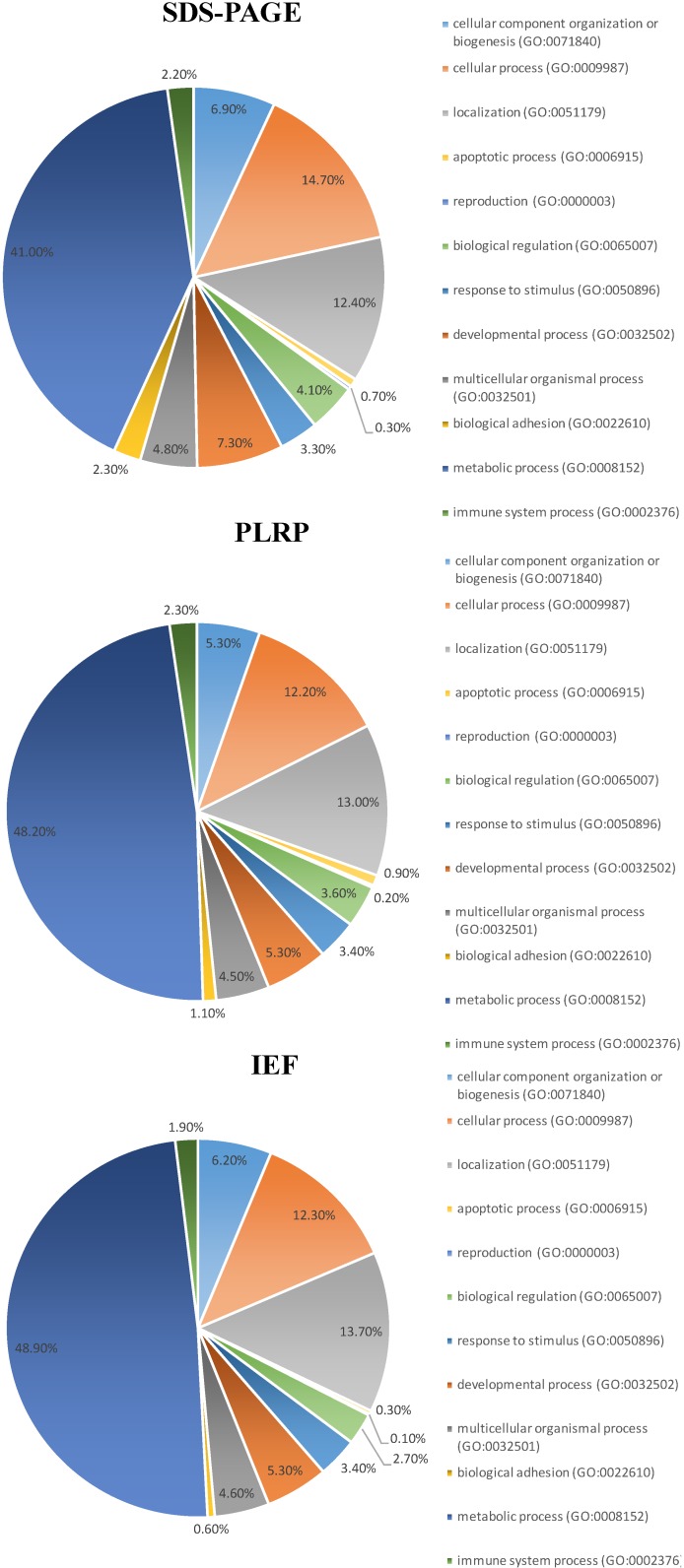
Gene ontology categorization of the identified proteins according to biological process.

To map the amino acid sequence motif distribution of the identified proteins we analyzed all proteins for *N*-terminal mitochondrial targeting sequences (MTS). Independently of the platform used, *ca.* 50% of all proteins identified contained MTS (Figure 5), according to TargetP [23]. It should be noted that many mitochondrial proteins do not contain an *N*-terminal MTS and are imported into the mitochondria through other mechanisms [24]. For cellular localization analysis of the identified proteins, ClueGO was used to determine the association strength between the Gene Ontology (GO) terms [25]. According to ClueGO, the majority of the identified proteins (61%) are part of different compartments related to mitochondria. Classification of the identified proteins by biological process was performed with the PANTHER bioinformatics tool (Figure 6) [26]. For all three platforms, the top cluster retrieved was metabolic processes (GO:0008152); between 41% and 49% of the identified proteins belong to this cluster, in keeping with the main function of the mitochondria in eukaryotic cells.

### 2.5. Selected Functional Characteristics of the Mitochondrial Proteome Panel

The mitochondrial proteome panel identified in the study includes proteins from various functional modules of the mitochondria. Information on proteins identified in selected functional clusters is presented in Table S4. In line with expectation, a significant proportion of the identified proteins belongs to the oxidative phosphorylation (OXPHOS) module. In total, 89 OXPHOS proteins were identified (Table S4), including subunits from all five complexes (I–V) that constitute the OXPHOS machinery. A second major functional class is components of the mitochondrial ribosome, with 39 members of this class present in our panel. A third functional category with significant representation includes proteins involved in protein translocation into the mitochondria; 17 members of the translocase of the outer membrane/translocase of the inner membrane (TIM/TOM) system were identified. Finally, the protein dataset contains several proteins that play an important role in mitochondrial fusion (e.g., dynamin-like 120 kDa protein—OPA1, and mitofusin-1 [27]), and two members of the protein deacetylase family (sirtuins 3 and 5 [28]).

### 2.6. Beyond Protein Identity—Protein Acetylation

**Figure 7:**
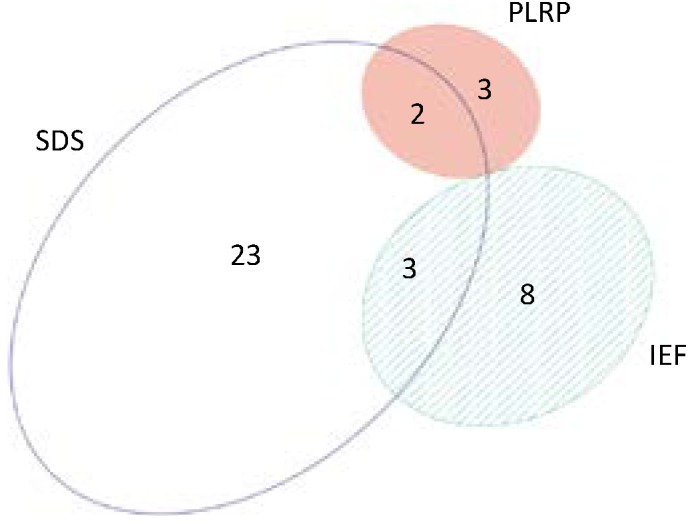
Characterization of protein acetylation in the mitochondrial proteome. The Venn diagram indicates the number of acetylated peptides characterized with each platform.

We searched the mitochondrial proteome datasets obtained in our study for the presence of post-translational modifications. Specifically, we focused on survey of acetylation of lysine residues in the mitochondrial proteins. It should be noted that enrichment steps to preferentially isolate modified proteins/peptides are commonly included in bioanalytical platforms used in studies of protein modifications. In this study, we have not included any enrichment steps, since the main focus was on proteome coverage (*i.e.*, protein identification). Nevertheless, in our non-enriched mitochondrial samples, a number of acetylated peptides/proteins were characterized. As shown in Figure 7, each bioanalytical platform yielded a set of acetylated peptides/sites. The largest number of acetylated peptides was found with the SDS-PAGE-based platform, in line with its superior performance for protein identification. A full list of the acetylated peptides is provided in Table S5; and their respective MS/MS spectra are provided in Figure S1. Novel lysine acetylation sites were identified in several mitochondrial proteins including proteins relevant to mitochondrial respiratory function such as electron transfer flavoprotein subunit α (P13803; Lys^301^), electron transfer flavoprotein subunit β (Q68FU3; Lys^200^), ATP synthase subunit d (P31399; Lys^63^), and ATP synthase subunit g (Q6PDU7, Lys^11^). The initial characterization of protein acetylation contributes to our ongoing efforts towards comprehensive description of post-translational modifications of the rat cardiomyocyte mitochondrial proteome [29].

## 3. Discussion

Examination of protein expression due to environmental, genetic or pharmacological perturbations on a global scale represents one of the most challenging tasks for the 21st century protein scientist. The introduction of ever improving analytical instrumentations and bioinformatics tools have allowed an unprecedented explosion of in-depth knowledge of complex biological systems. The study of the mitochondrion represents one of the most promising fields of biochemical investigation, which might bring new insight in the mechanisms of natural processes such as aging, pathophysiological mechanisms in diseases such as Alzheimer’s, Parkinson’s, and heart failure, and health impacts caused by environmental stressors such as alcohol and cigarette smoking [1]. To be able to gain insights in all these complex biochemical events, system-wide analytical tools are indispensable.

In our research on the pathophysiological mechanisms that characterize the failing heart, we use a rat model of heart failure to investigate the mitochondrial proteome perturbations associated with the applied experimental conditions [30]. To be able to effectively probe the mitochondrial proteome at its deepest possible level, an optimal analytical platform needs to be chosen [31,32]. We have compared three widely used analytical platforms: PLRP [33], IEF [14,34] and SDS-PAGE [35]. All three methods are simple to implement, need no specialized, high-cost equipment and are highly effective to fractionate complex protein samples. The choice of the number of fractions in the first dimension (*i.e.*, 15) is a good balance between the protein mapping coverage and the need for a practical sample throughput [14]. The results of this study unequivocally demonstrate the superior performance of the SDS-PAGE-based platform. The better performance of this platform can be explained, at least partially, by the better protein solubility and protein denaturation features associated with the use of SDS. Although, the IEF method also uses chaotropic agents to facilitate protein solubilization, the SDS strong protein denaturation capabilities allow for a more complete post-gel proteolytic processing. In addition, the higher pore size of the IEF gel strips as compared to the SDS-PAGE gels increases the losses of proteins from the gel plugs during sample preparation, which typically involve multiple washing steps. Compared to the PLRP method, both the IEF and SDS-PAGE platforms had a higher protein count and higher protein coverage. We believe that the better performance of the gel-based platforms relates to a more efficient sample proteolysis, facilitated by the protein denaturation caused by the chaotropic agents used for protein solubilization prior to electrophoresis and by incomplete sample recovery from the PLRP column.

## 4. Experimental Section

### 4.1. Mitochondria Isolation and Protein Extraction

Experimental procedures for the use of animals were approved by our Institutional Animal Care and Use Committee (11-038.0, 24 January 2012). Mitochondria were obtained from 8-week-old Sprague-Dawley rats (*n* = 6). The subsarcolemmal population of mitochondria was isolated from freshly collected heart tissue by differential centrifugation as previously described. Briefly, heart tissue samples were homogenized, and the homogenates were processed via differential centrifugation. IFM were discarded after 1500× *g*, and the SSM harvested at 10,000× *g*. The purity of mitochondrial preparations was assessed by flow cytometry and mitochondria-specific dye, Mito Tracker Red (Invitrogen, Carlsbad, CA, USA), as previously described [18].

Prior to proteome analyses, individual purified mitochondrial preparations were pooled and proteins were extracted with the aid of sonication. After extraction, total protein was quantified with the 2D-Quant protein assay kit (GE Healthcare, Piscataway, NJ, USA). The whole protein extract was divided into three equal portions, and stored at −80 °C until further processing. A total of 400 µg of mitochondrial protein was used for analyses with each bioanalytical platform; from this amount, 130 µg were used in each technical replicate (*n* = 3 per platform).

### 4.2. Isoelectric Focusing (IEF)

IEF was performed with the Multiphor II unit (GE Healthcare) as previously described [14]. In brief, mitochondrial proteins were dissolved in an IEF rehydration buffer containing urea (7 M), thiourea (2 M), 3-[(3-cholamidopropyl)dimethylammonio]-1-propanesulfonate (CHAPS) detergent (2% *w*/*v*), immobilized pH gradient (IPG) buffer (carrier ampholyte mixture; 2% *v*/*v*), and dithiothreitol (DTT, 0.3% *w*/*v*). Eighteen-cm-long IPG strips with a nonlinear 3–10 pH gradient were used for IEF. The protein samples (*ca.* 130 µg per strip) were applied via passive IPG strip rehydration. Focusing was performed according to the following protocol: 0–100 V (gradient over 1 min); 100 V (fixed for 120 min); 100–500 V (gradient over 1 min); 500–3500 V (gradient over 90 min); 3500 V (fixed for 8 h). Following IEF, the proteins were fixed with trichloroacetic acid (20% *w*/*v*) [14], and with a solution of CH_3_OH–H_2_O–CH_3_COOH, 50:40:10 (*v*:*v*:*v*). Each IPG strip was divided into 15 sections of equal size.

### 4.3. Sodium Dodecyl Sulfate-Polyacrylamide Gel Electrophoresis (SDS-PAGE)

For SDS-PAGE, *ca.* 130 µg of mitochondrial protein was used in each technical replicate. This protein amount was loaded in three gel lanes (40 µg per lane), which were then combined during further processing. SDS-PAGE was performed with pre-cast Criterion XT 4%–12% Bis-Tris gels, 13.3 × 8.7 cm (Biorad, Hercules, CA, USA). Each gel was developed at 200 V for 60 min. The proteins were visualized with Coomassie Blue stain, and each gel lane was divided into 15 equal-size sections.

### 4.4. In-Solution Protein Digestion

Four hundred micrograms of mitochondrial protein was digested and divided into three equal aliquots for PLRP separations at the peptide level. In brief, the proteins were digested with sequencing grade modified trypsin (Promega, Madison, WI, USA) in 50 mM ammonium bicarbonate buffer (ABC) at a ratio of protease-to-substrate of 1:50, for 18 h at 37 °C. After digestion, the solution was acidified with trifluoroacetic acid (TFA), and the peptides were desalted by solid-phase extraction (SPE) with a home-made spin minicolumn filled with *ca.* 200 mg of Vydac C18 packing material (Sigma-Aldrich, St. Louis, MO, USA). The column-bound peptides were washed with 500 µL of H_2_O (0.1% TFA), repeated 3 times. Peptides were eluted with 400 µL of CH_3_CN–H_2_O, 70:30 (*v*/*v*, 0.1% TFA) and stored at −80 °C until use.

### 4.5. In-Gel Protein Digestion

Proteins in IEF strip sections and SDS-PAGE gel sections were in-gel digested with trypsin. Prior to digestion, the proteins were reduced with DTT (50 mM in 200 mM ABC) and alkylated with iodoacetamide (110 mM in 200 mM ABC). The gel pieces were dehydrated with acetonitrile and dried in a vacuum centrifuge. The dried gels were rehydrated with a digestion solution containing sequencing-grade trypsin (Promega) in 50 mM ABC; the protease-to-protein ratio was *ca.* 1:50. The digestion was carried out overnight at 37 °C. After digestion, the peptide mixtures were acidified with TFA, centrifuged, and the supernatants were collected. To recover remaining peptides, the gel pieces were incubated with 100 µL of CH_3_CN–H_2_O–TFA, 60:35:5 (*v*:*v*:*v*), centrifuged, and the supernatants were combined with the original digests. All samples were dried in a vacuum centrifuge. Prior to LC-MS/MS analysis, each sample was subjected to C_18_ extraction using ZipTip minicolumns (Millipore, Billerica, MA, USA) following manufacturer’s procedure. The peptides were eluted from the ZipTip with 4 µL of CH_3_CN–H_2_O 50:50 (*v*/*v*, 0.1% TFA) and diluted with 6 µL of 0.1% formic acid.

### 4.6. High-pH Reversed-Phase Chromatography (PLRP)

The purified peptide solution volume was reduced to *ca.* 10 µL and mixed with 10 µL of PLRP mobile phase buffer A (H_2_O:NH_4_OH (30%); (99.8:0.2, *v*:*v*), pH 10). Twenty microliters of peptide solution were manually injected onto polymer-based reversed phase column (PLRP-S, 5 µm, 100 Å, 150 × 2.1 mm) from Agilent Technologies (Santa Clara, CA, USA) connected to an HPLC system (Varian Model 9012 pump and UV detector Model 9050, Agilent, Santa Clara, CA, USA). Peptides were eluted with a linear gradient from 0% B (CH_3_CN:H_2_O:NH_4_OH (30%); (80:19.8:0.2, *v*:*v*:*v*), pH 10) to 90% B over a period of 95 min, followed by 5 min isocratic elution, 5 min re-equilibration (90%–0% B) and 10 min isocratic, prior to the next injection. Column flow rate was set at 0.2 mL/min. Peptide elution was monitored by UV detection at 230 nm wavelength. Peptides were fractionated with an HPLC fraction collector (Spectra/Chrom, Model CF-1, Spectrum Chromatography, Houston, TX, USA). Fifteen fractions per run were collected. The peptides solutions were dried in a vacuum centrifuge, re-suspended in 10 µL H_2_O, 0.1% TFA and stored at −80 °C until ready for analysis.

### 4.7. Liquid Chromatography-Mass Spectrometry (LC-MS/MS)

The analyses were performed with an LTQ ion trap mass spectrometer (Thermo Fisher Scientific, Waltham, MA, USA) interfaced with a Famos/Ultimate nano LC system (Dionex, Sunnyvale, CA, USA). Two microliters of peptide digests were manually injected onto a nano injector (Idex, Oak Harbor, WA, USA). Peptides were separated on a home-packed capillary column (100 mm × 75 µm I.D.) (New Objective, Woburn, MA, USA) packed with Magic C_18_ (5 µm, 200 Å, Michrom Bioresources, Auburn, CA, USA). The peptides were eluted at a flow rate of 300 nL/min with a 95 min linear gradient from solvent A ((H_2_O:CH_3_OH:HCOOH), (97.9:2:0.1), *v*:*v*:*v*) to solvent B ((H_2_O: CH_3_OH:HCOOH), (9.9:90:0.1), *v*:*v*:*v*). The mass spectrometer was operated in a data-dependent scan mode, where a survey MS scan (400–2000 *m*/*z*) was followed by MS/MS of the seven most intense precursor ions with dynamic exclusion on.

### 4.8. Bioinformatics

The raw data were input into Proteome Discoverer version 1.4 (Thermo Fisher Scientific, Waltham, MA, USA) and searched with Sequest HT against a rat database (Swiss-Prot; number of protein sequences 28,885; version 06/27/2013) *in silico* digested with trypsin. The search parameters were: full tryptic specificity, precursor error tolerance 1.5 Da, product ion tolerance 0.8 Da. Allowed peptide modifications were carbamidomethylation of cysteine (static modification), and methionine oxidation and lysine acetylation (dynamic modifications). To calculate the false discovery rate for matched peptides, the same rat reversed database was used as a decoy and the resulting data were statistically analyzed by Percolator, a new standard component of Proteome Discoverer [19,20]. Only peptides with PEP scores of 0.01 or lower were considered as positive matches.

The GRAVY peptide and protein scores were calculated based on the hydrophobicity amino acid parameters as described by Kyte and Doolittle [36] implemented in an Excel (Microsoft) script. All data were analyzed with Prism (v5.0.4) from GraphPad (La Jolla, CA, USA).

Gene Ontology (GO) annotations were analyzed with the PANTHER Protein Classification System [37] to identify functional annotations. To predict the subcellular location of the identified proteins, the data were analyzed with TargetP [38] and the ClueGO [25] plug-in from Cytoscape (v3.0.2 [39]) and applying the Gene Ontology rat database. Information on protein function was compiled from Swiss-Prot annotations and primary literature.

## 5. Conclusions

Comparison of three different gel-based and gel-free platforms demonstrated that protein separation by SDS-PAGE in combination with LC-MS/MS is the most powerful bioanalytical strategy for mitochondrial proteome profiling. The SDS-PAGE-based platform allowed an in-depth characterization of the rat subsarcolemmal mitochondrial proteome. A total of 890 proteins were identified, most with very high sequence coverage, including proteins relevant to mitochondrial bioenergetics, protein import, mitochondrial fusion and other critical functional aspects. This study generated one of the largest inventories of rat cardiomyocyte mitochondrial proteins, which can be used as foundation for the design of quantification studies to characterize perturbations of the mitochondrial proteome in the context of heart failure and other diseases.

## Supplementary Files

Supplementary File 1 Supplementary Materials (ZIP, 1080 KB)
