# Supplementary material for: Proteome Analysis of Subsarcolemmal Cardiomyocyte Mitochondria: A Comparison of Different Analytical Platforms
Source: Int J Mol Sci. 2014 May 26;15(6):9285–301. doi: 10.3390/ijms15069285 (PMC4100094; doi:10.3390/ijms15069285)
Supplement: Supplementary File 1 — Supplementary Materials (ZIP, 1080 KB) [file ijms-15-09285-s001.zip › ijms-54611-Figure S1 for publication.pdf]

Supplementary Information

Figure S1. Mass Spectrometry (MS/MS) spectra for acetylated peptides. SDS-PAGE, sodium dodecyl sulfate-polyacrylamide gel electrophoresis; IEF, isoelectric focusing; PLRP, polymeric reversed-phase liquid chromatography at high pH. (**1**–**28**) From SDS-PAGE; (**29**–**36**) From IEF; (**37**–**39**) From PLRP.

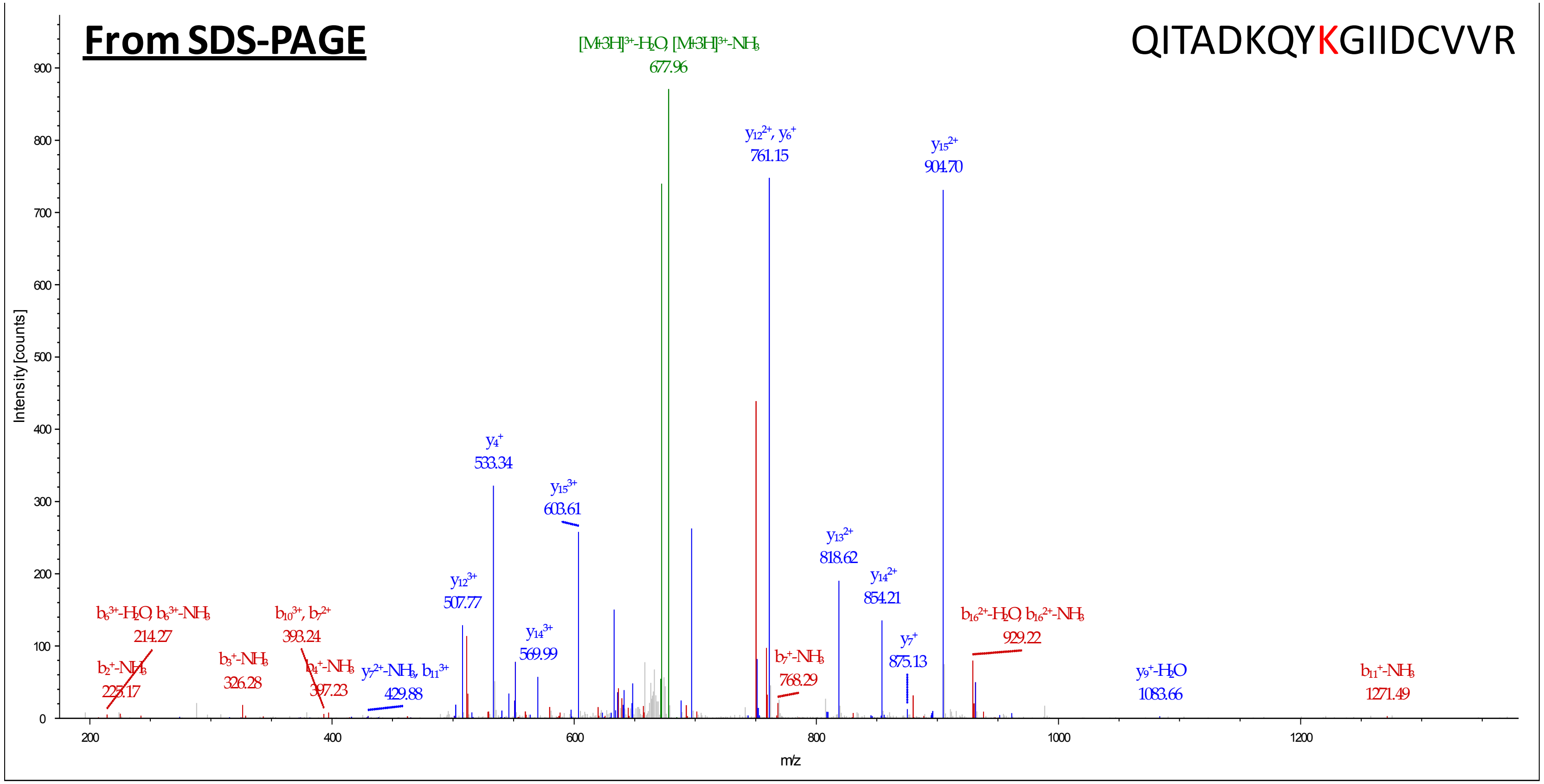

(1)

Figure S1. *Cont.*

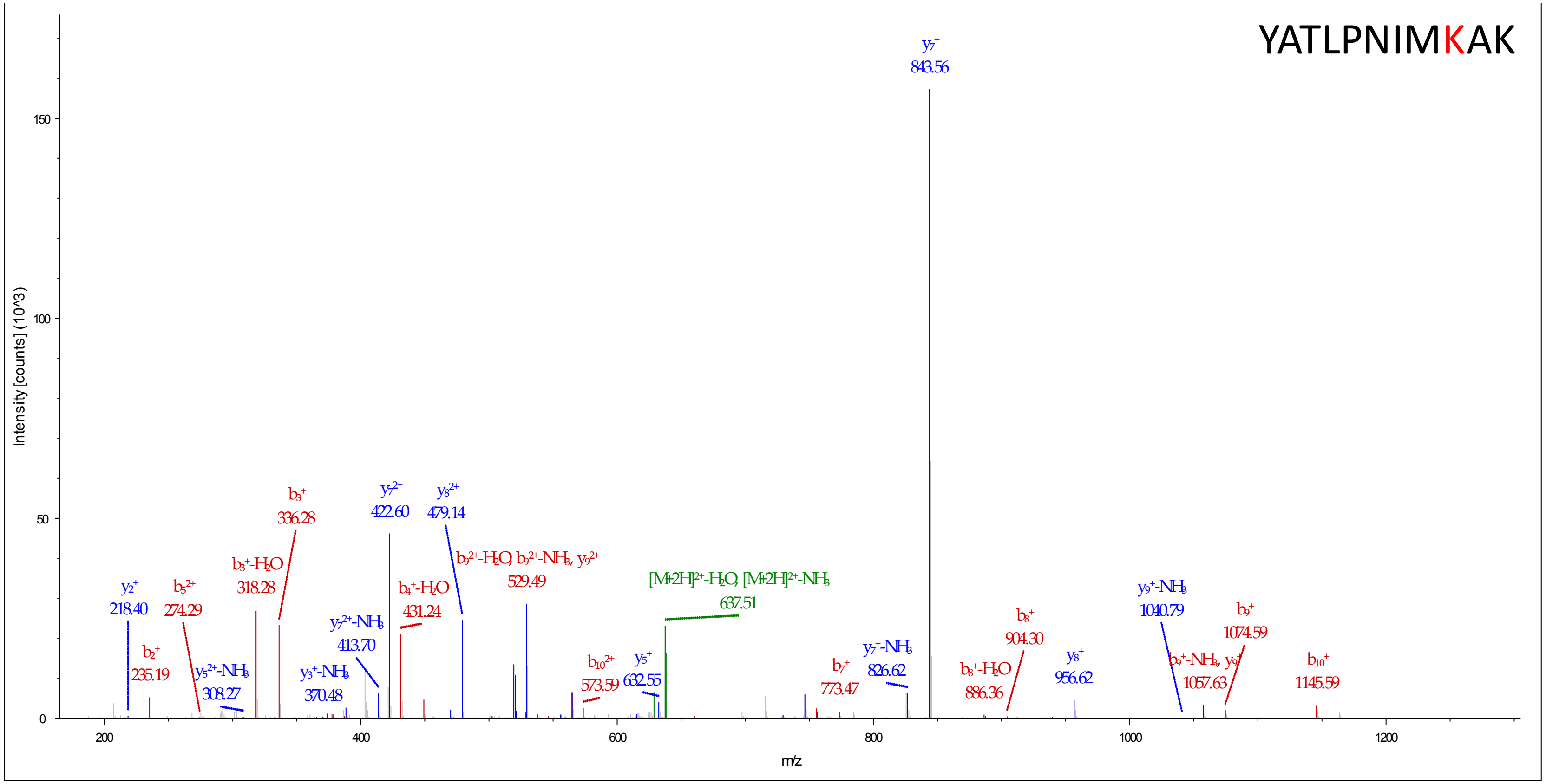

(2)

Figure S1. *Cont.*

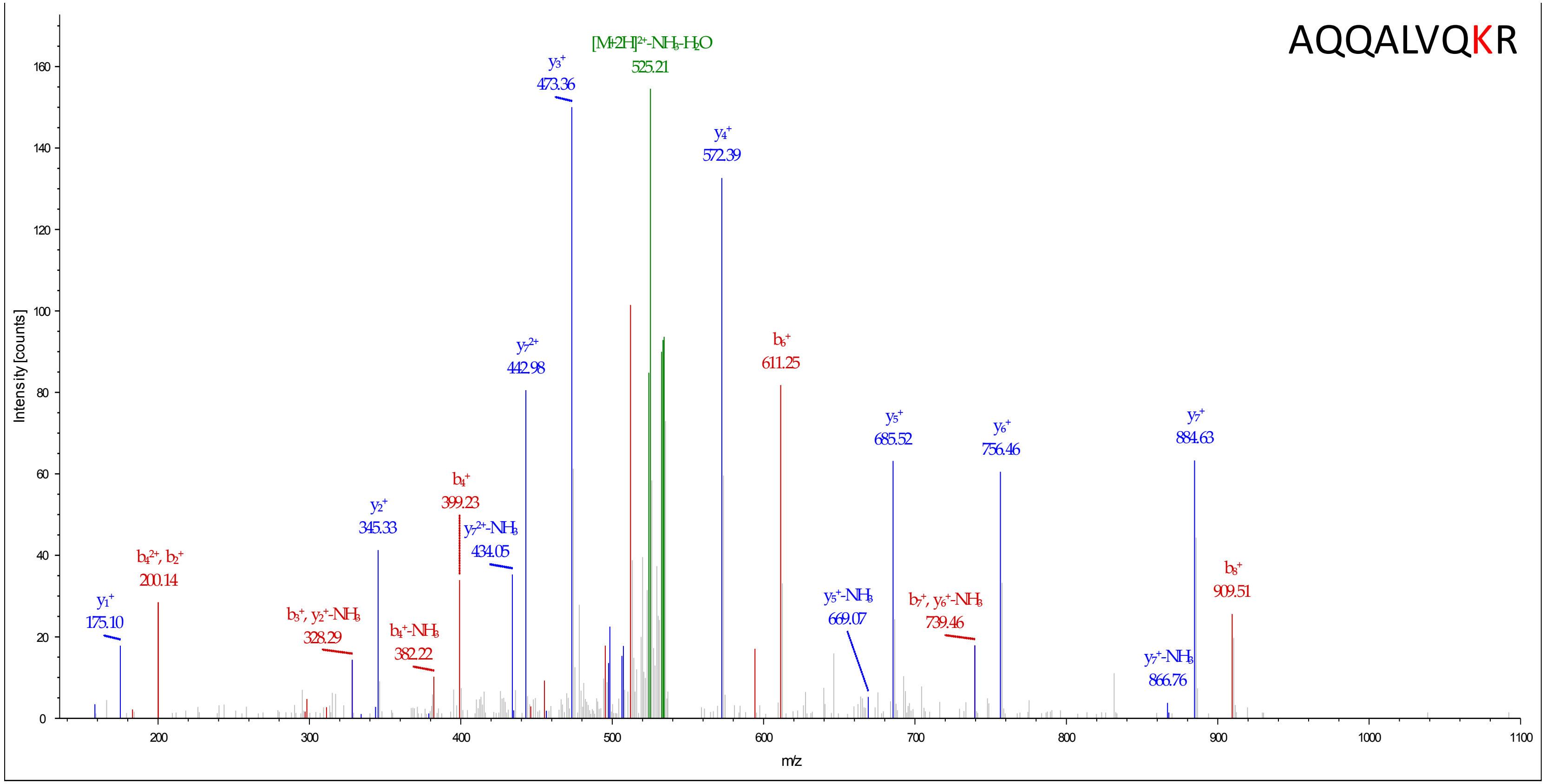

(3)

Figure S1. *Cont.*

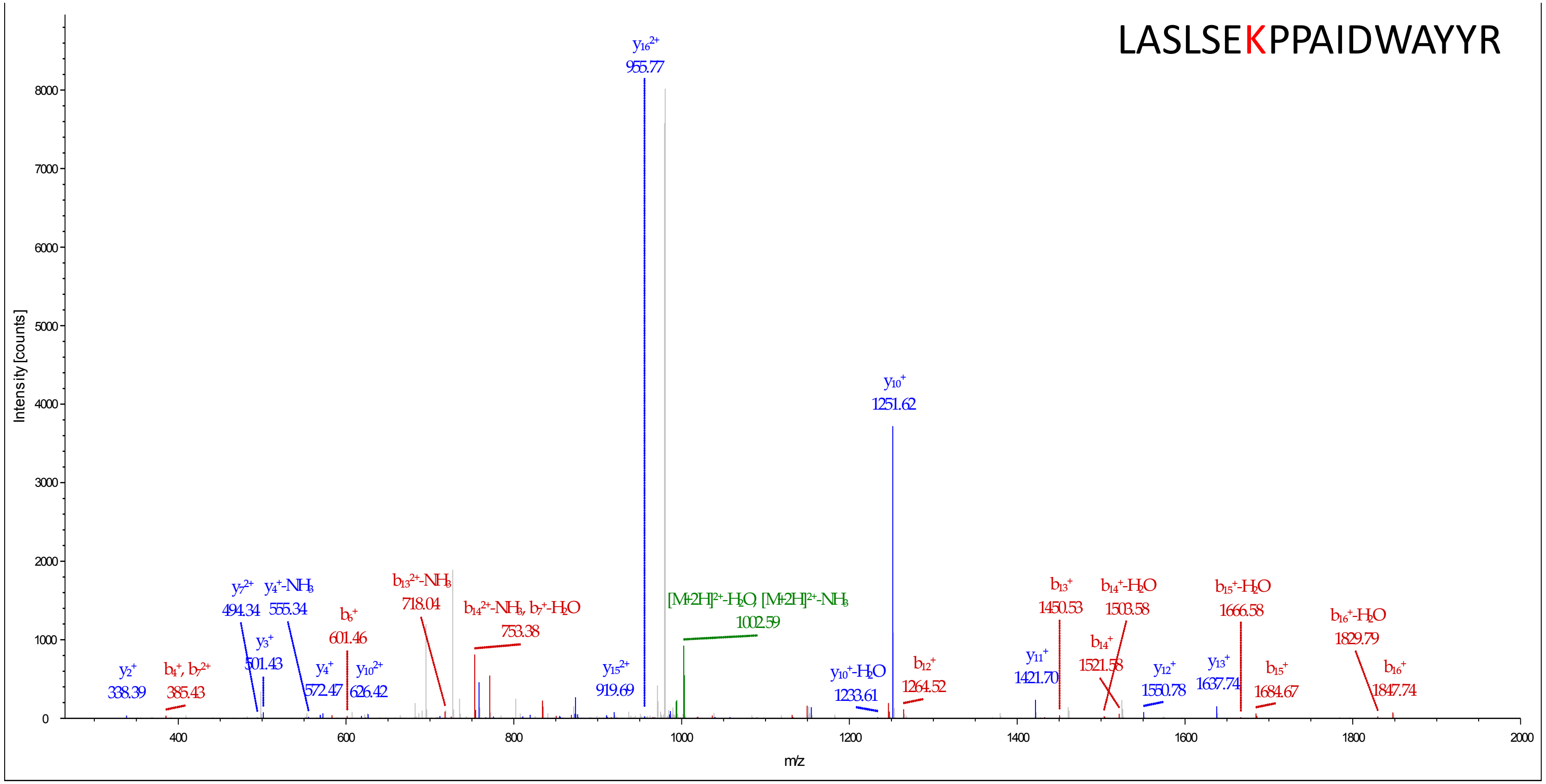

(4)

Figure S1. *Cont.*

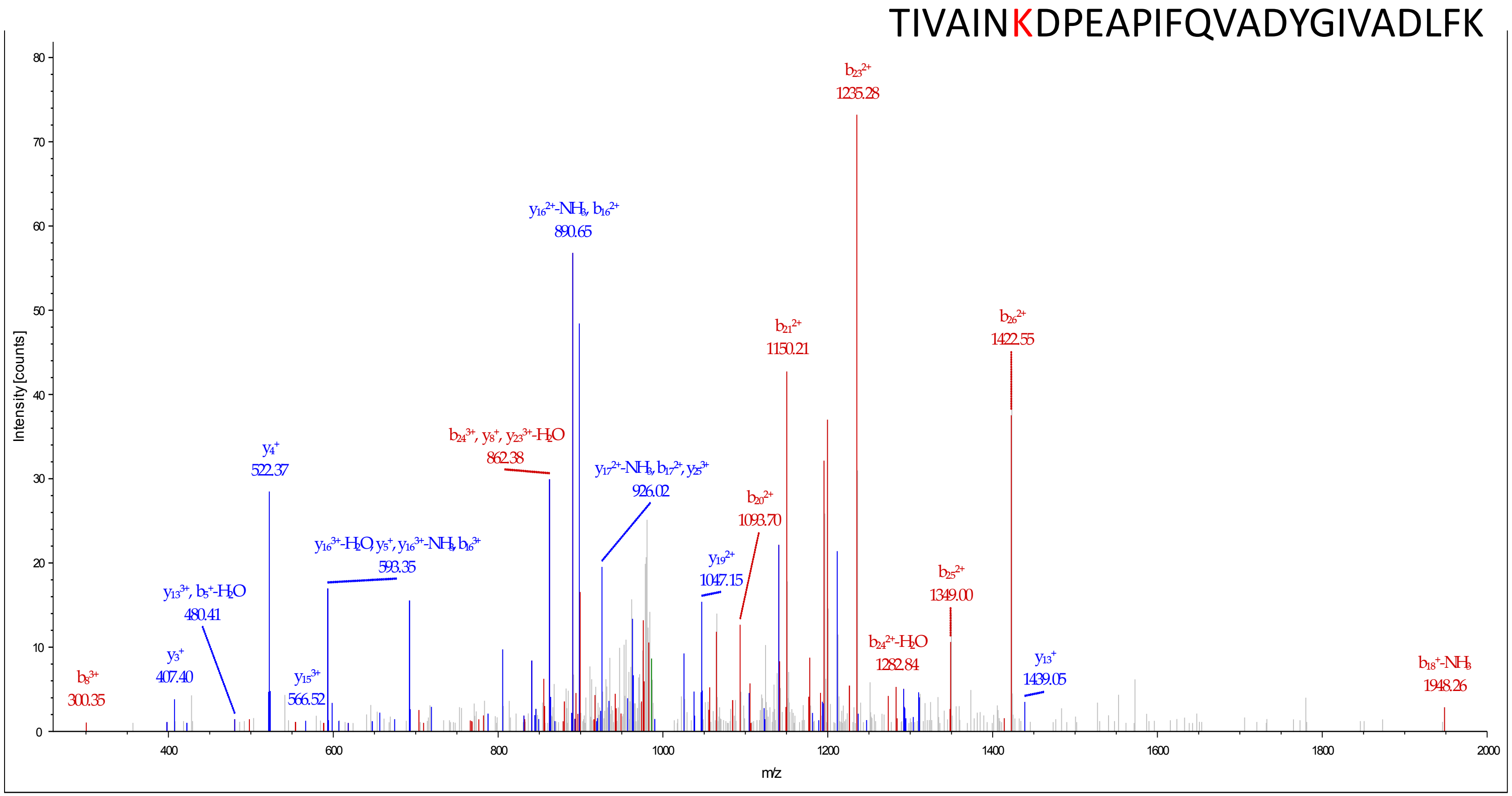

(5)

Figure S1. *Cont.*

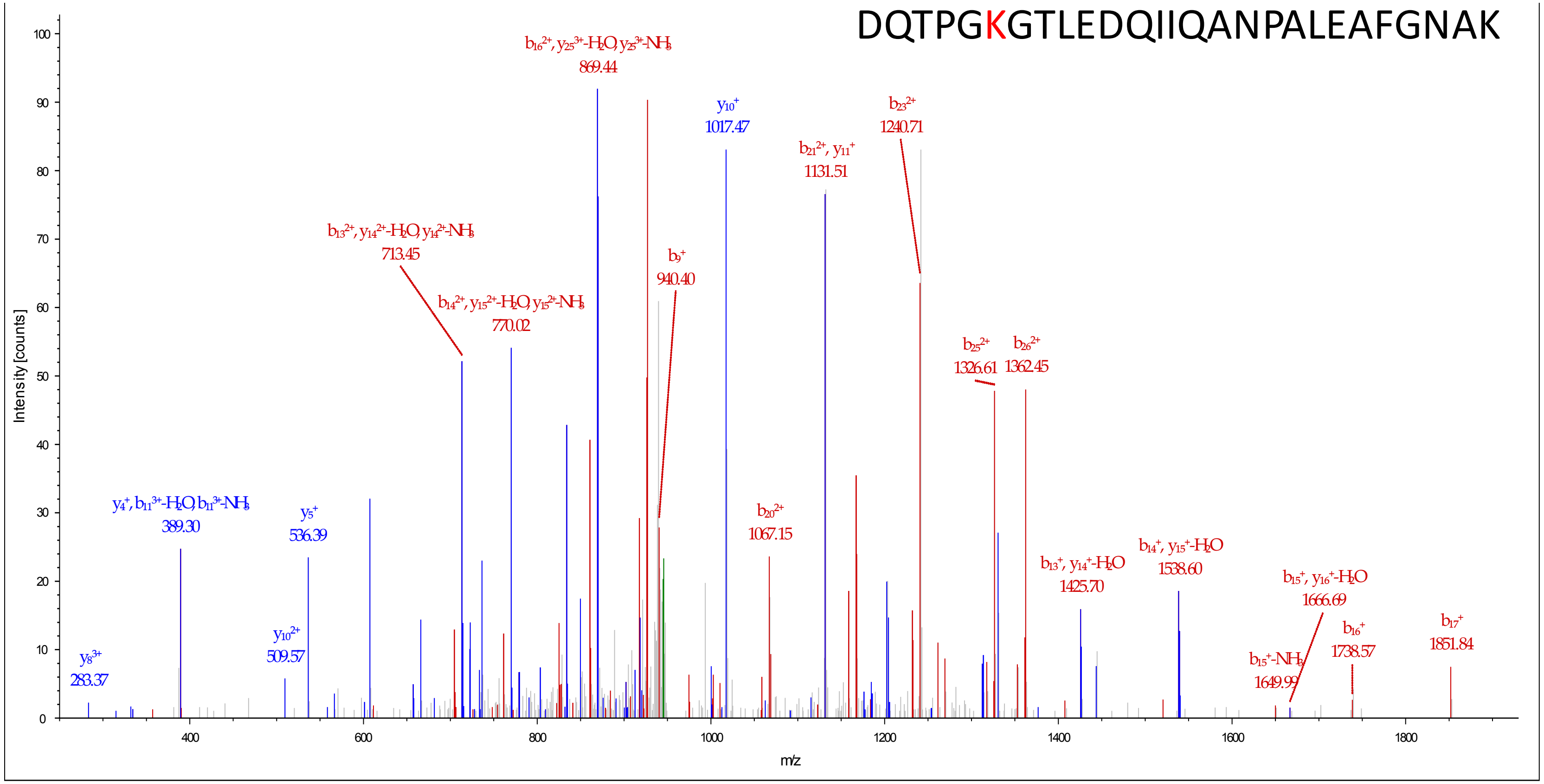

(6)

Figure S1. *Cont.*

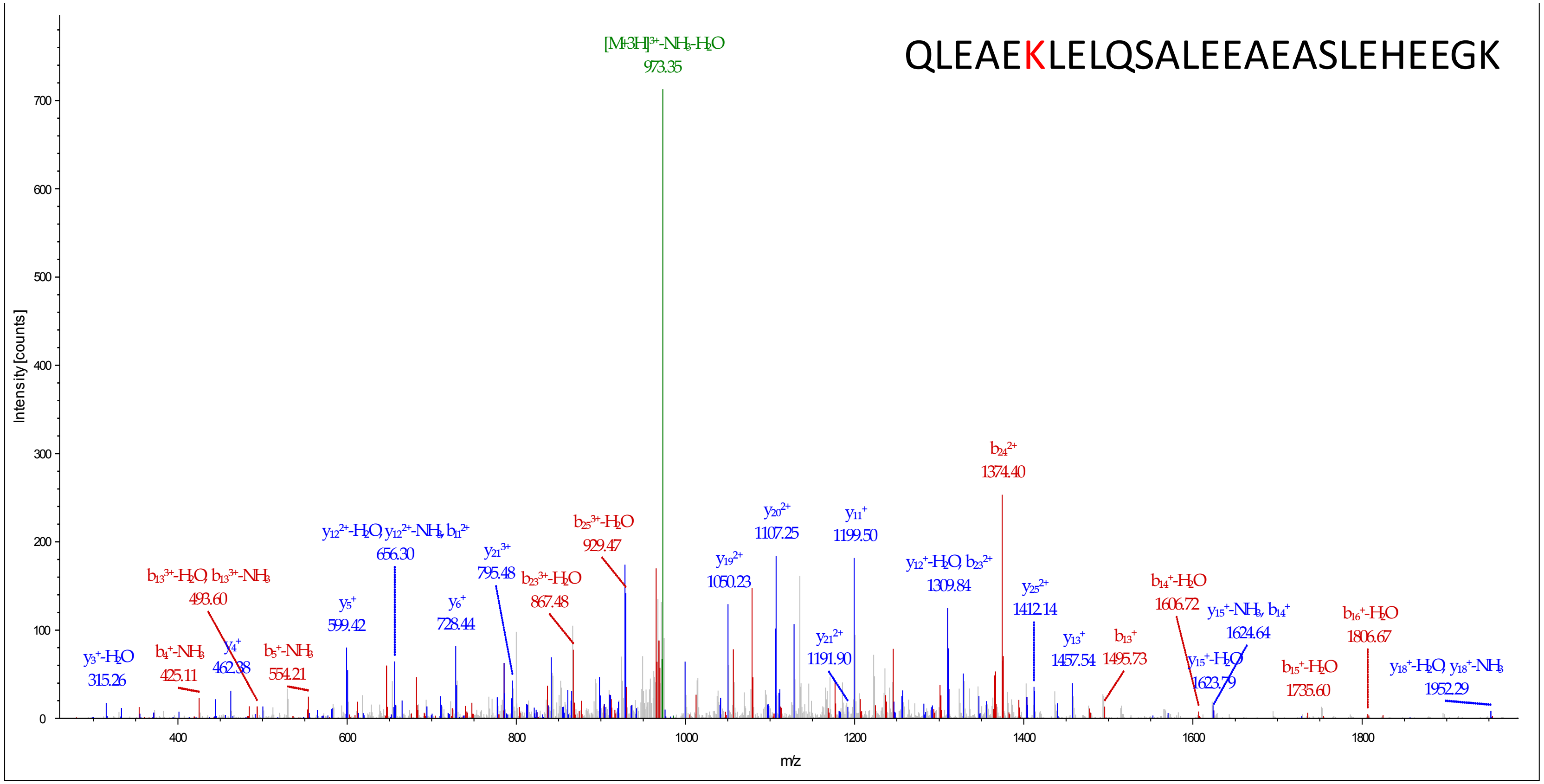

(7)

Figure S1. *Cont.*

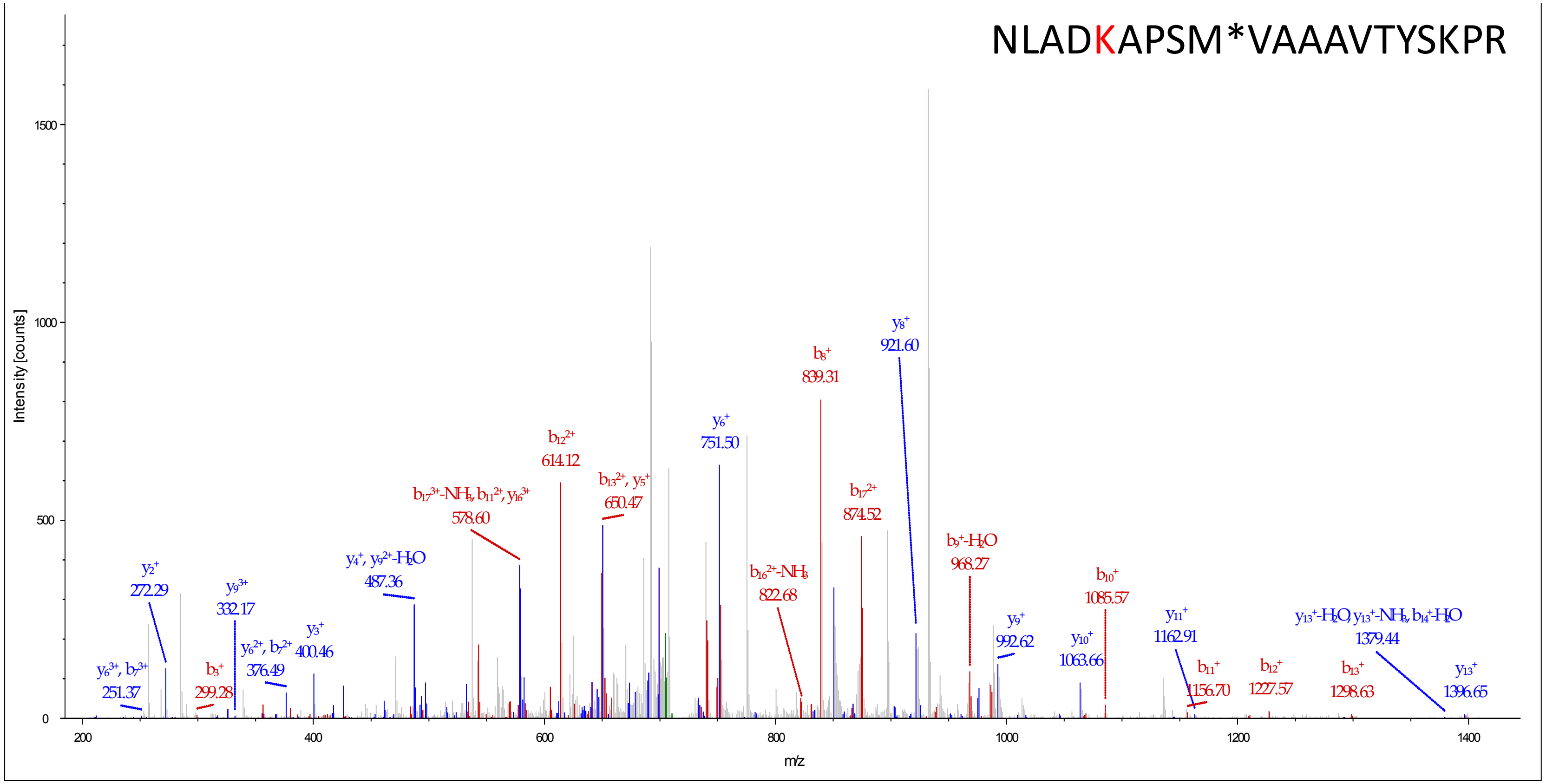

(8)

Figure S1. *Cont.*

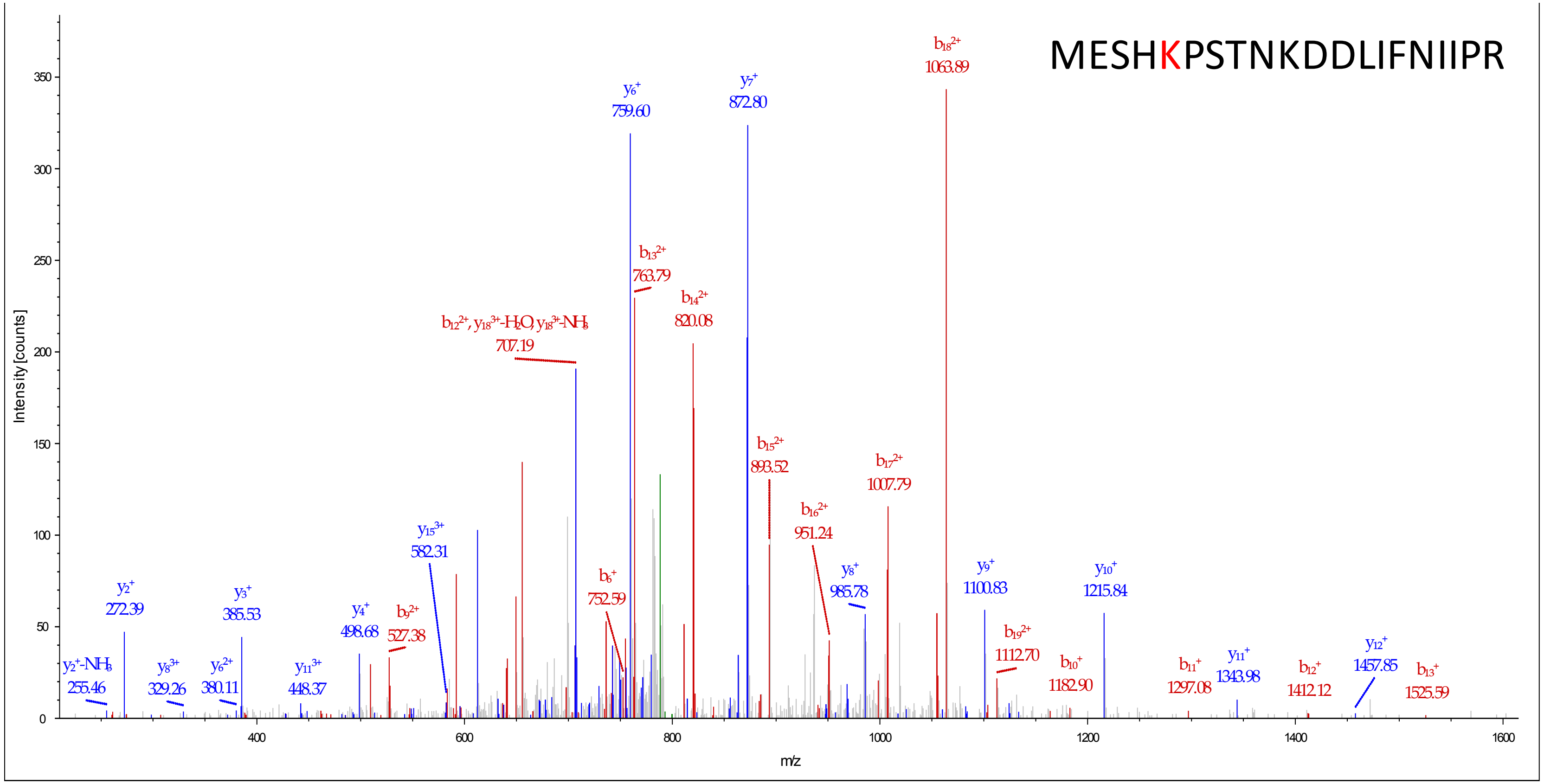

(9)

Figure S1. *Cont.*

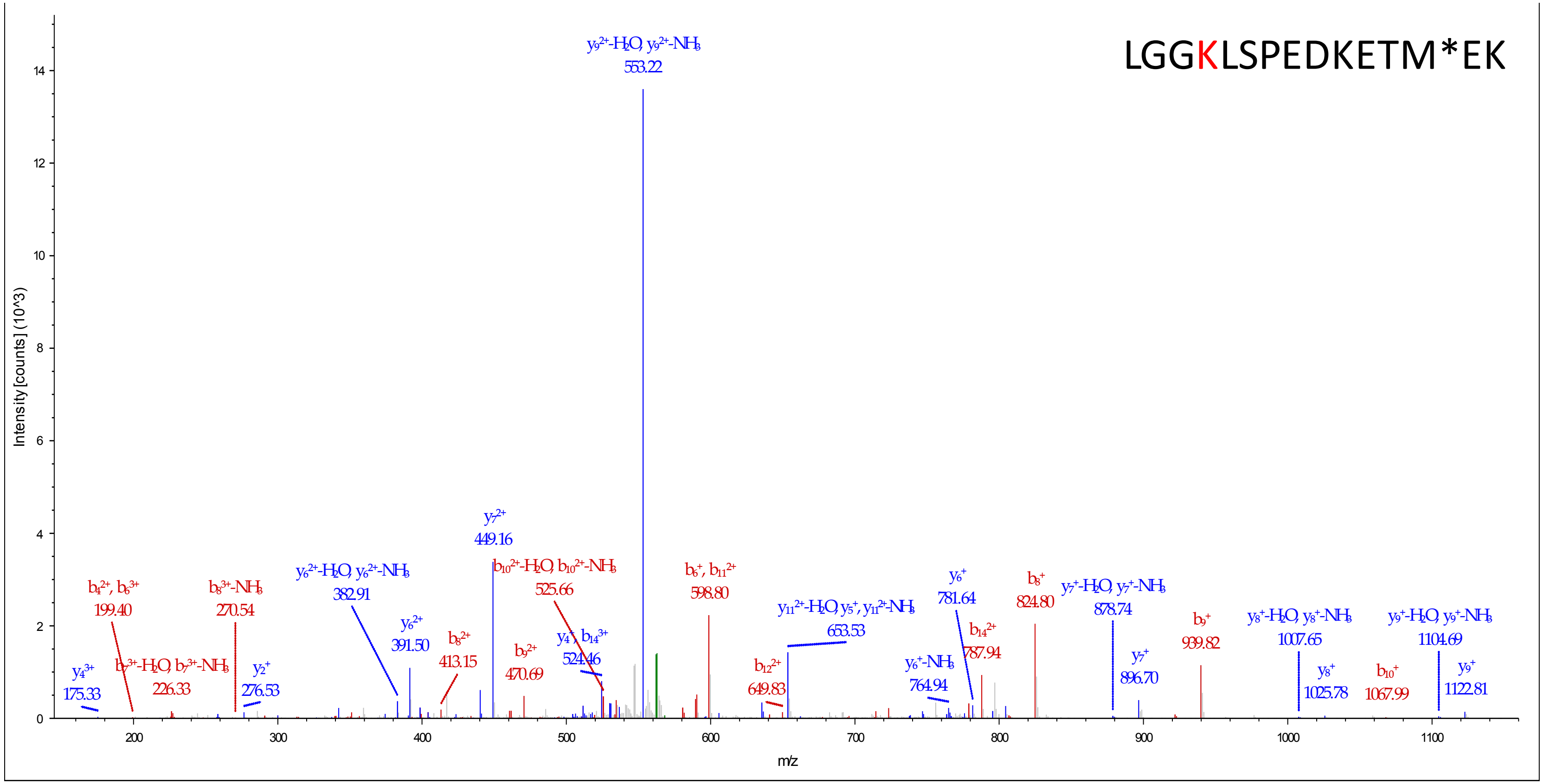

(10)

Figure S1. *Cont.*

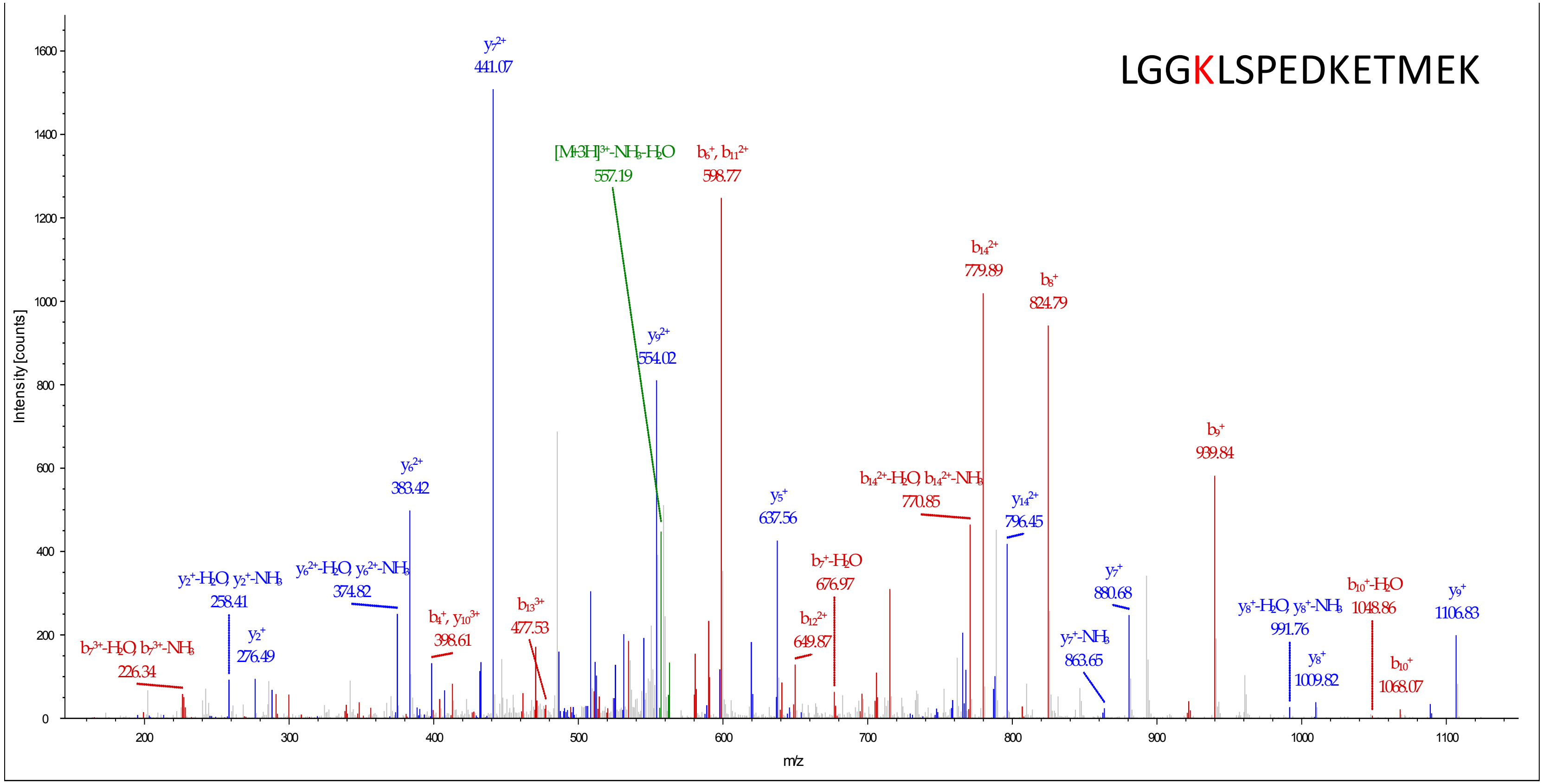

(11)

Figure S1. *Cont.*

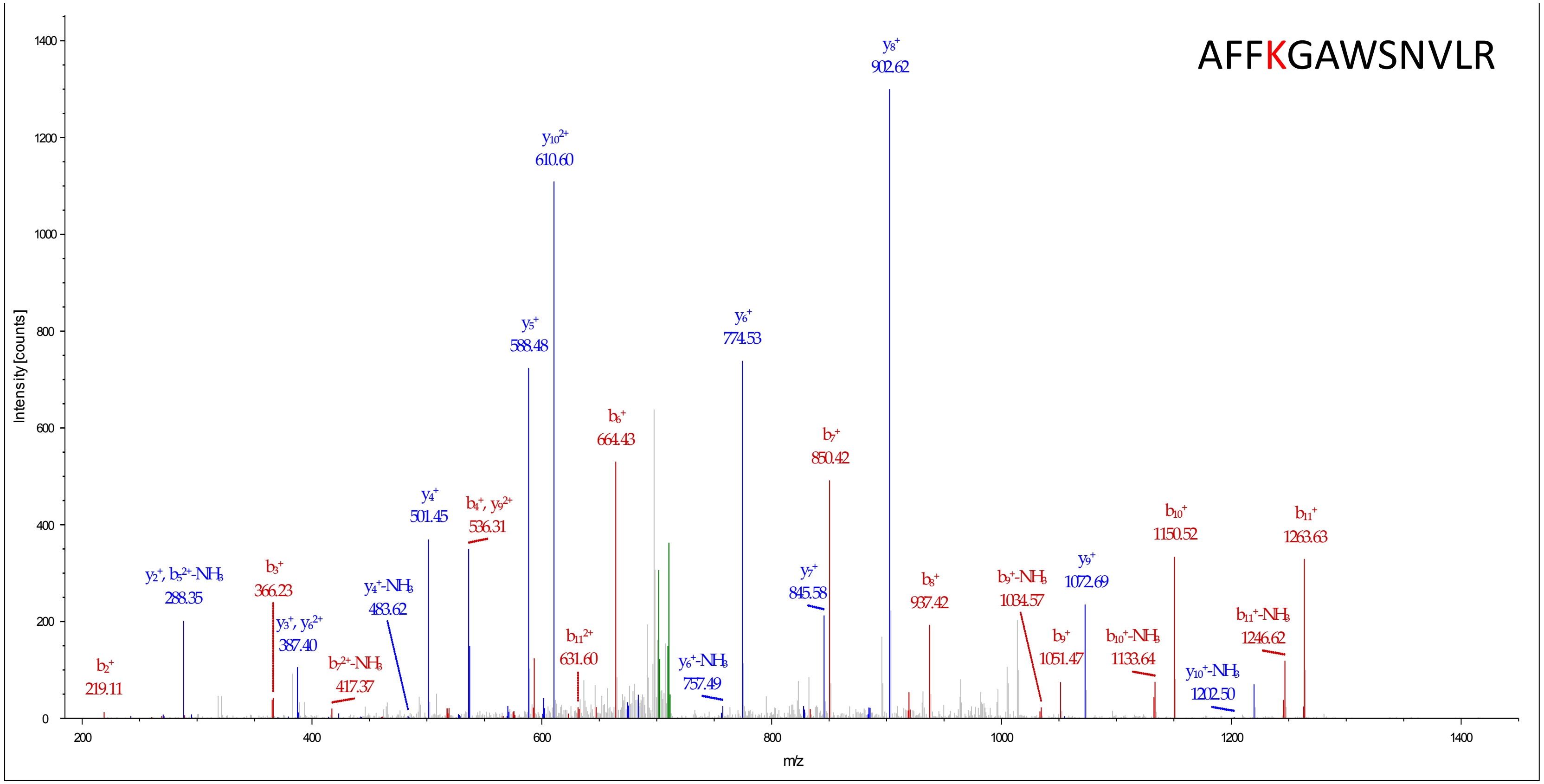

(12)

Figure S1. *Cont.*

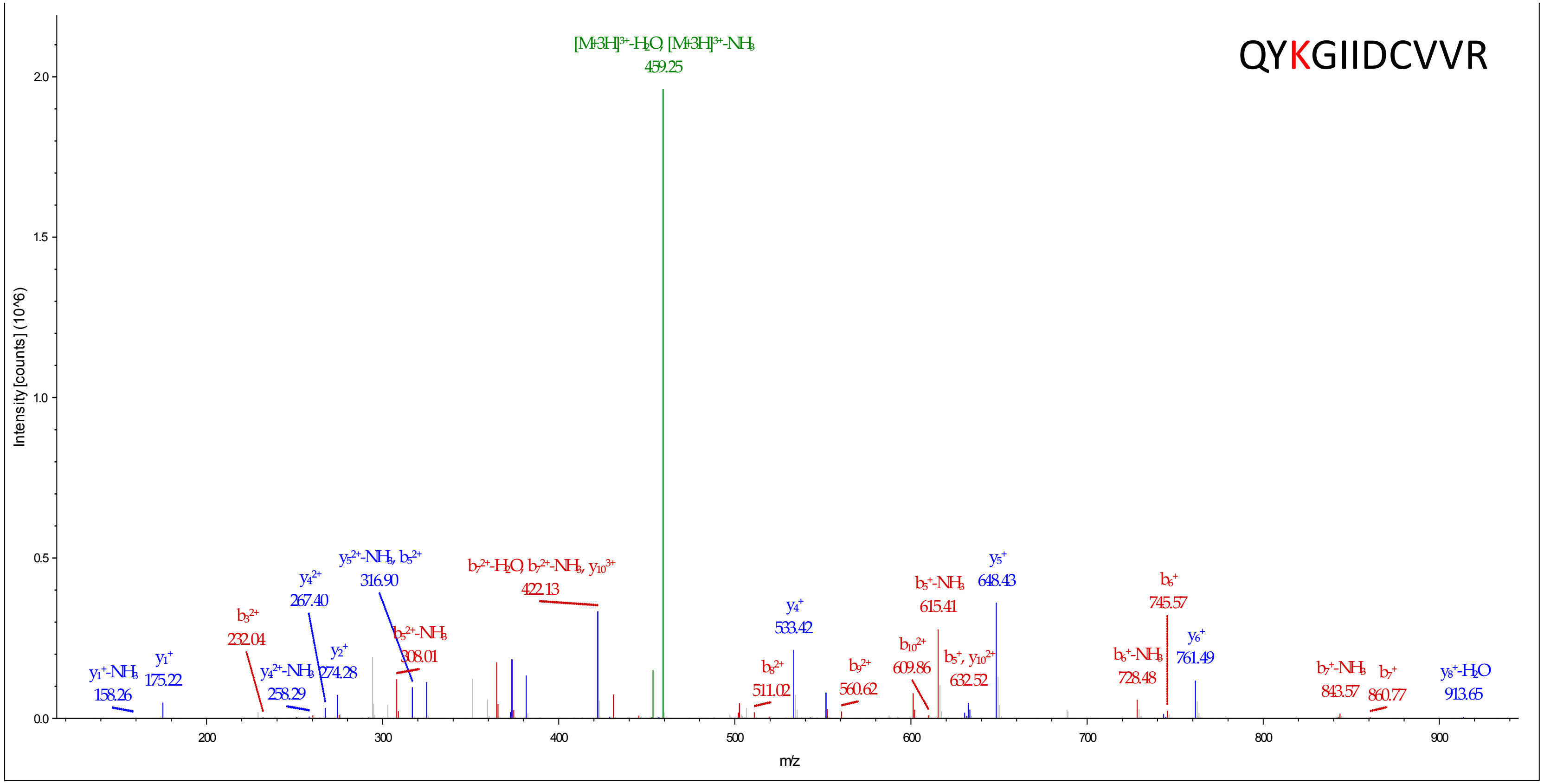

(13)

Figure S1. *Cont.*

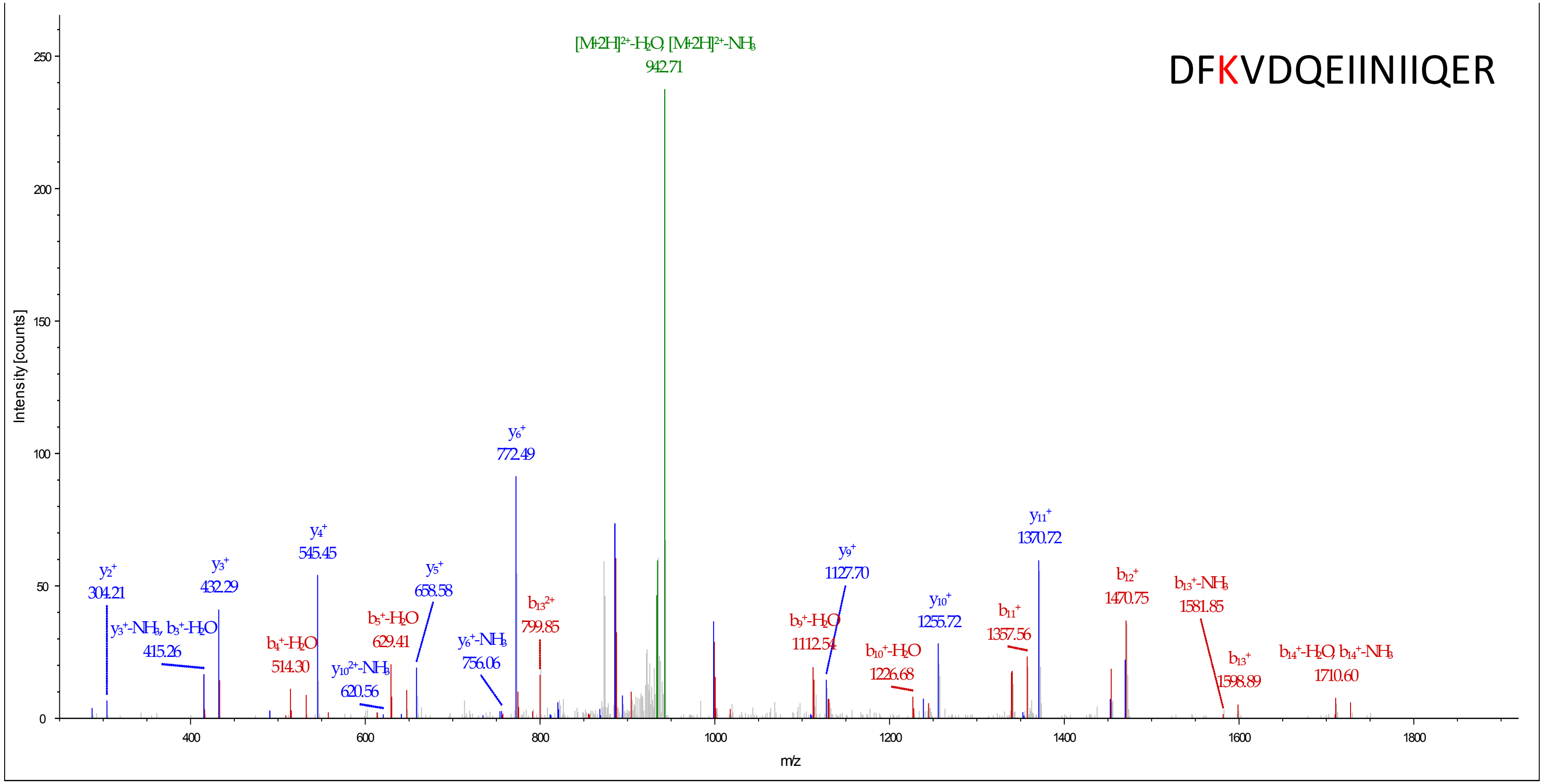

(14)

Figure S1. *Cont.*

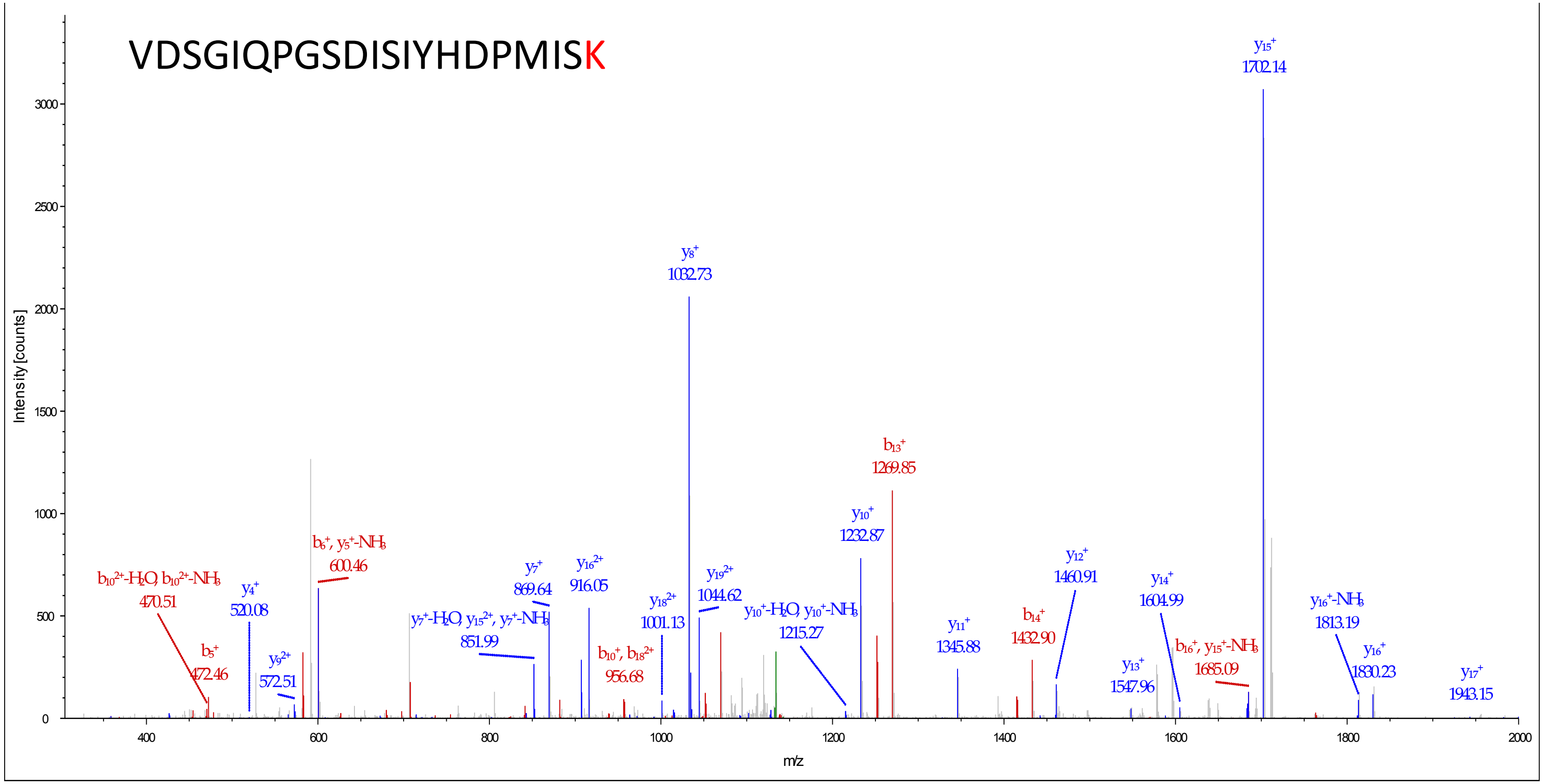

(15)

Figure S1. *Cont.*

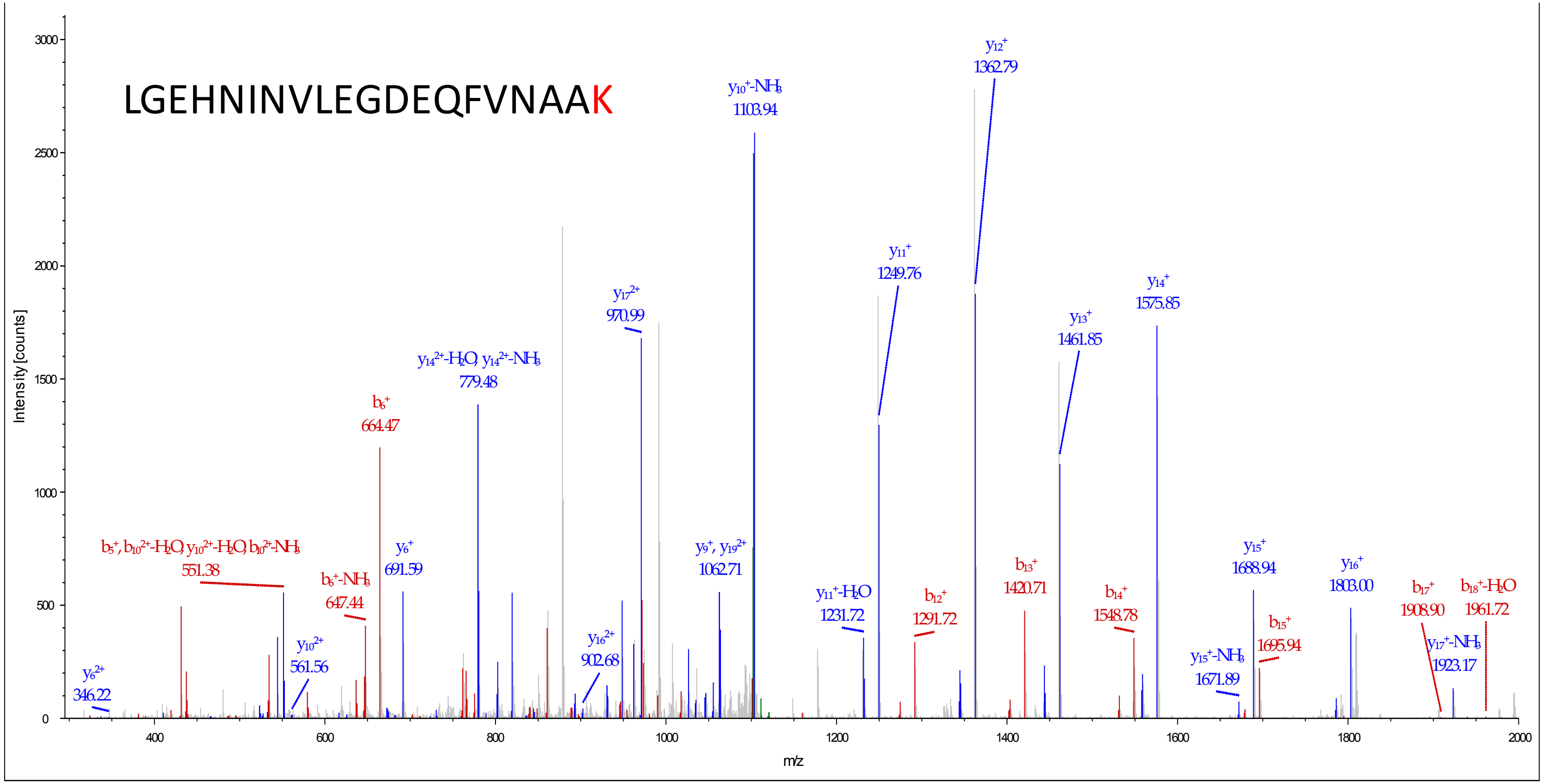

(16)

Figure S1. *Cont.*

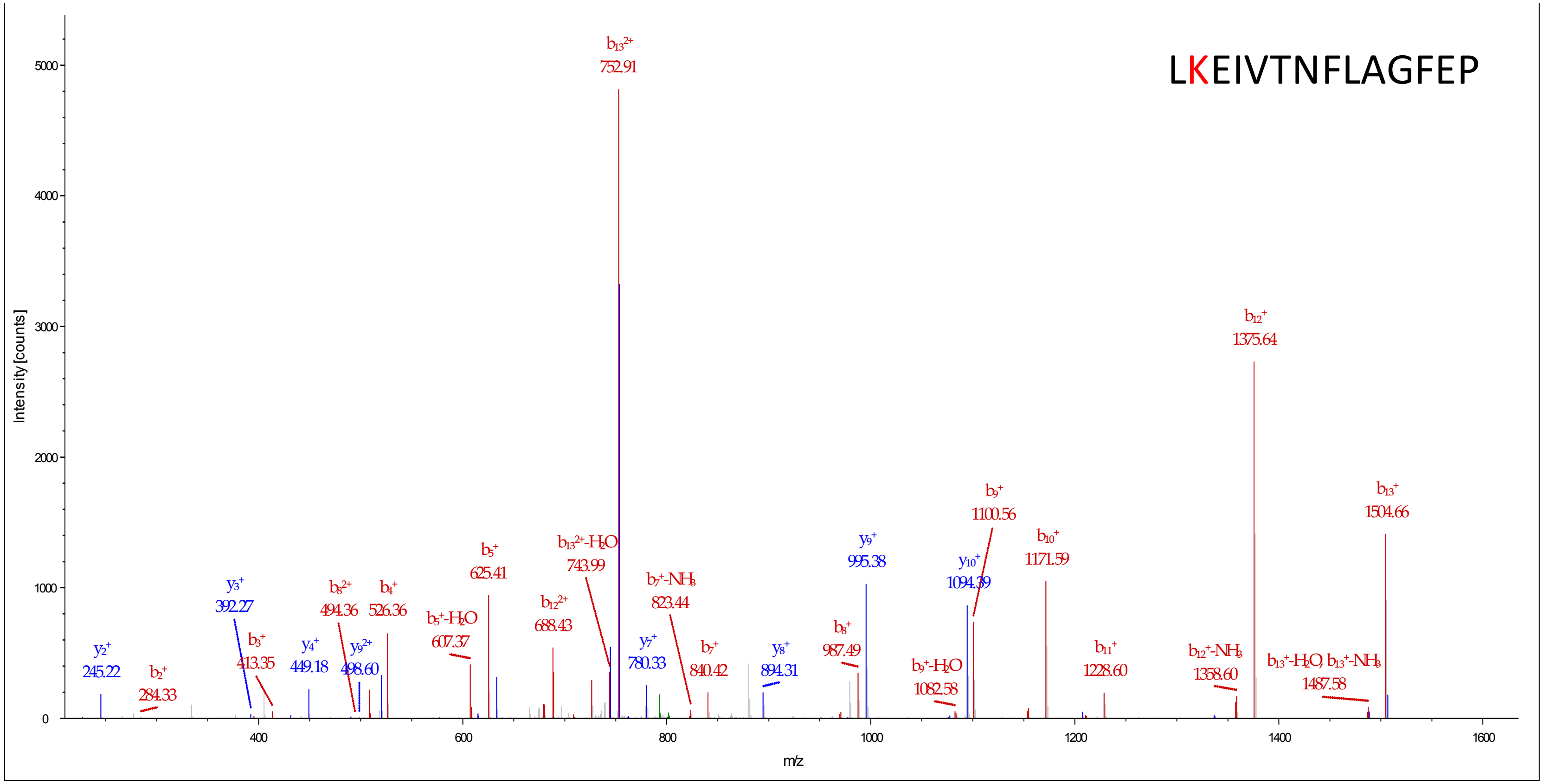

(17)

Figure S1. *Cont.*

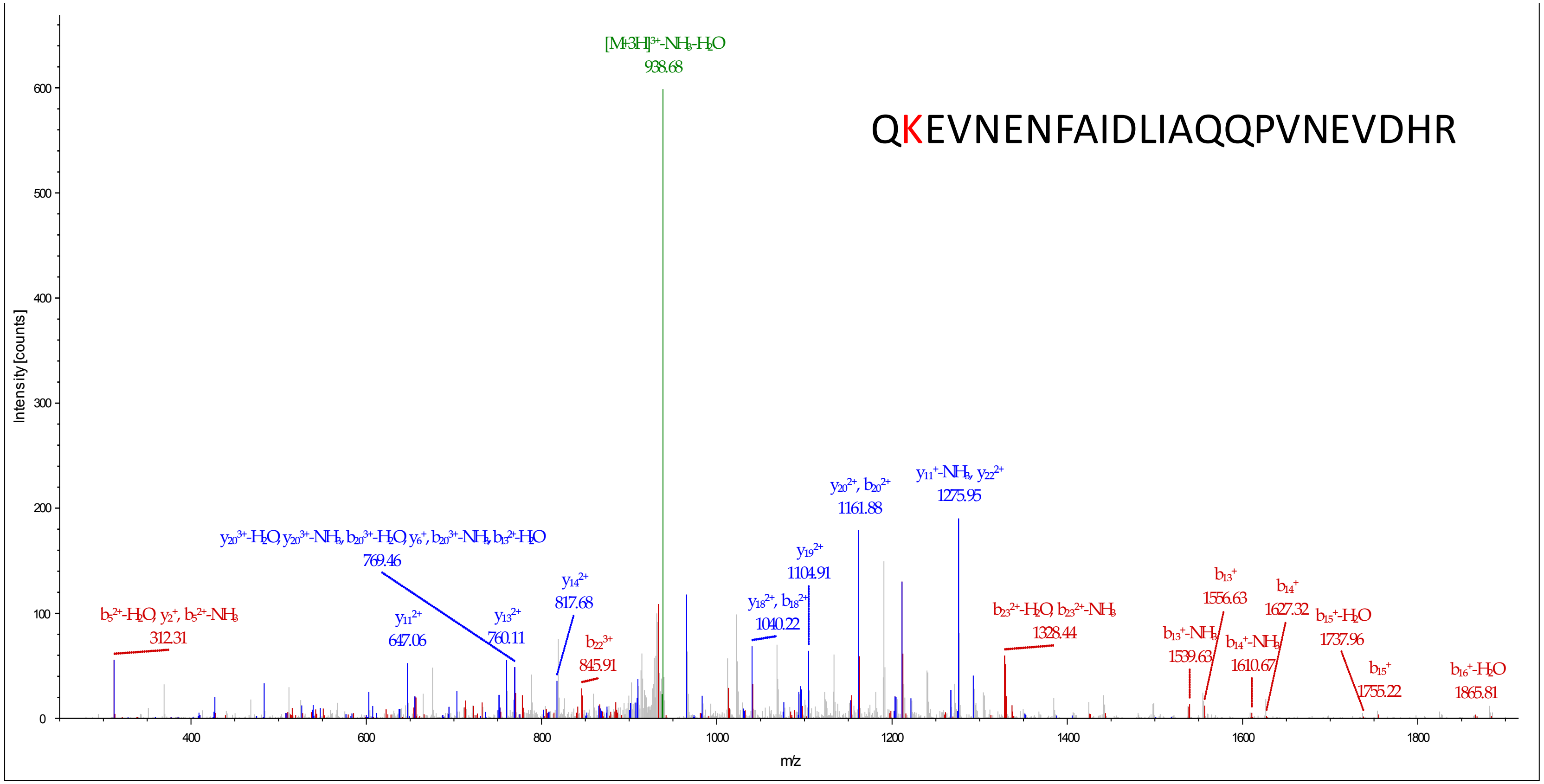

(18)

Figure S1. *Cont.*

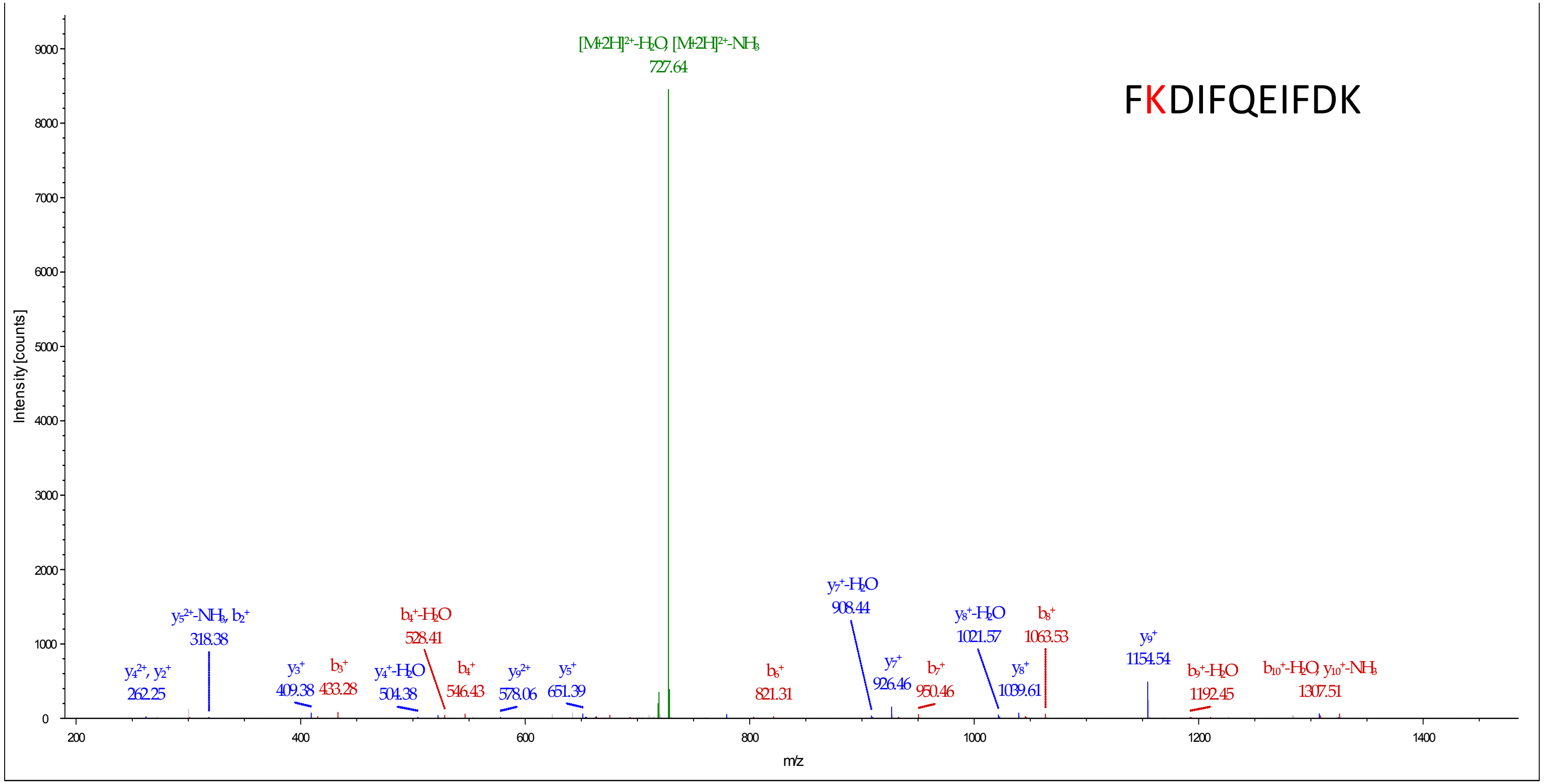

(19)

Figure S1. *Cont.*

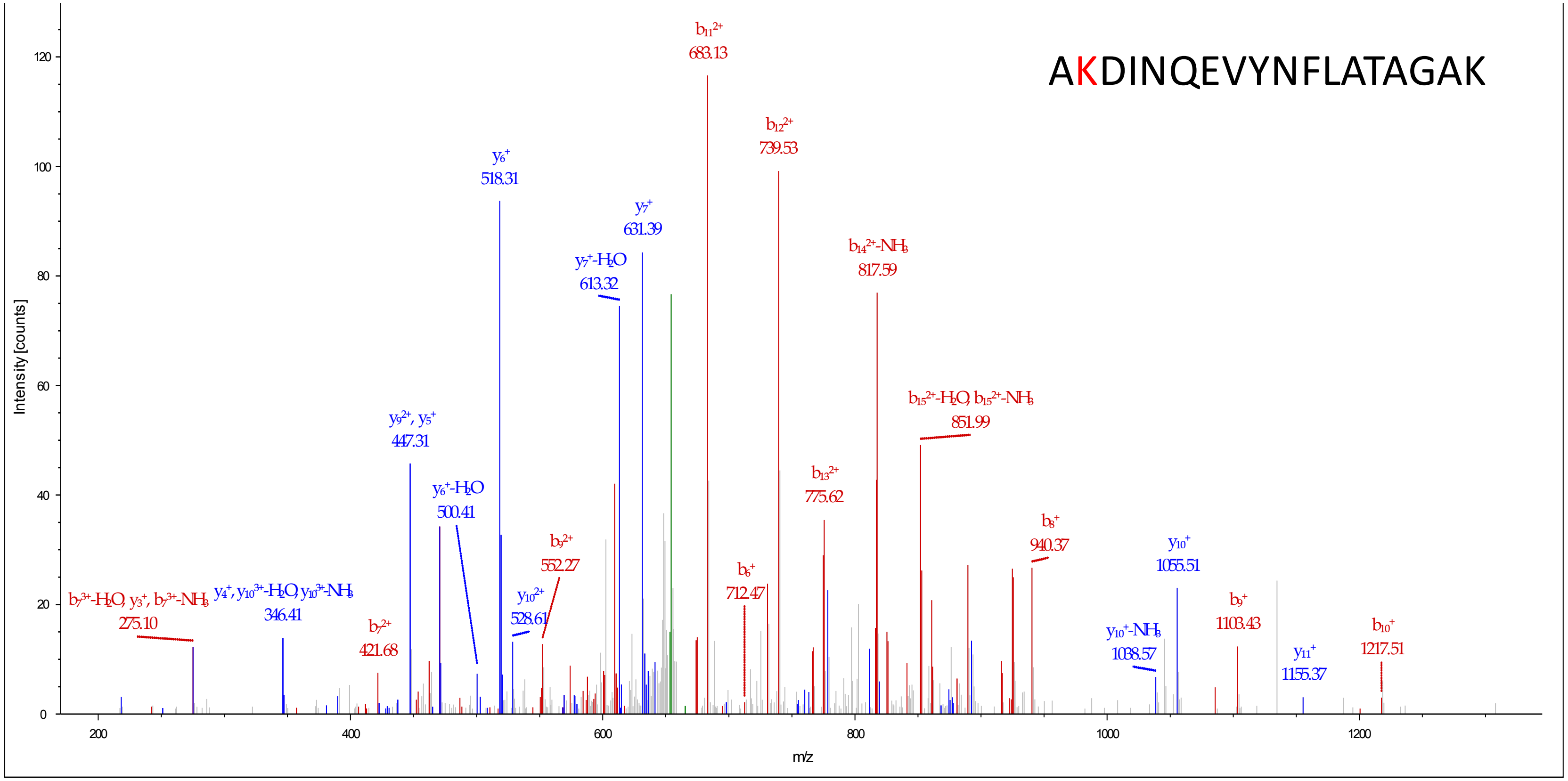

(20)

Figure S1. *Cont.*

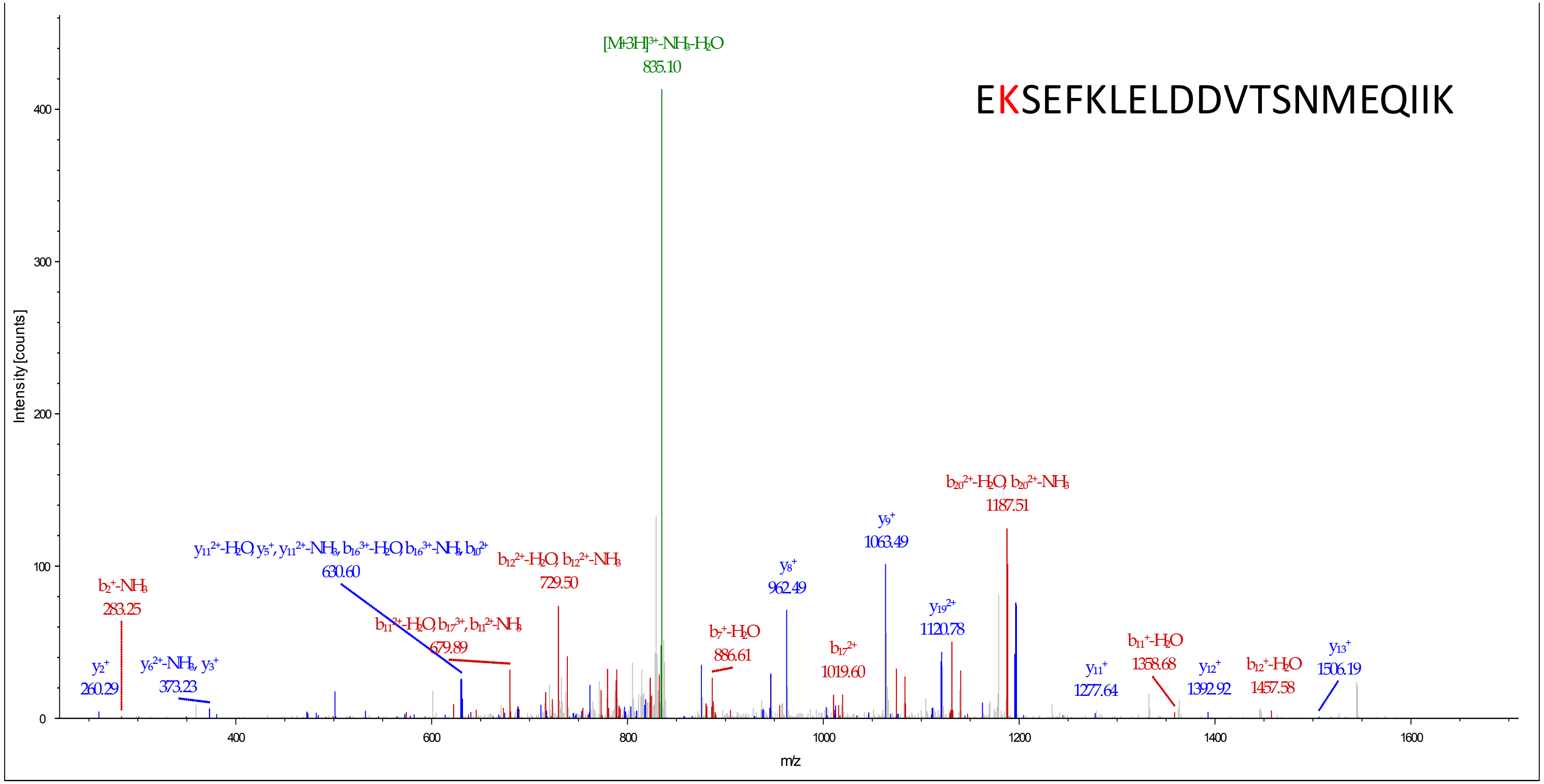

(21)

Figure S1. *Cont.*

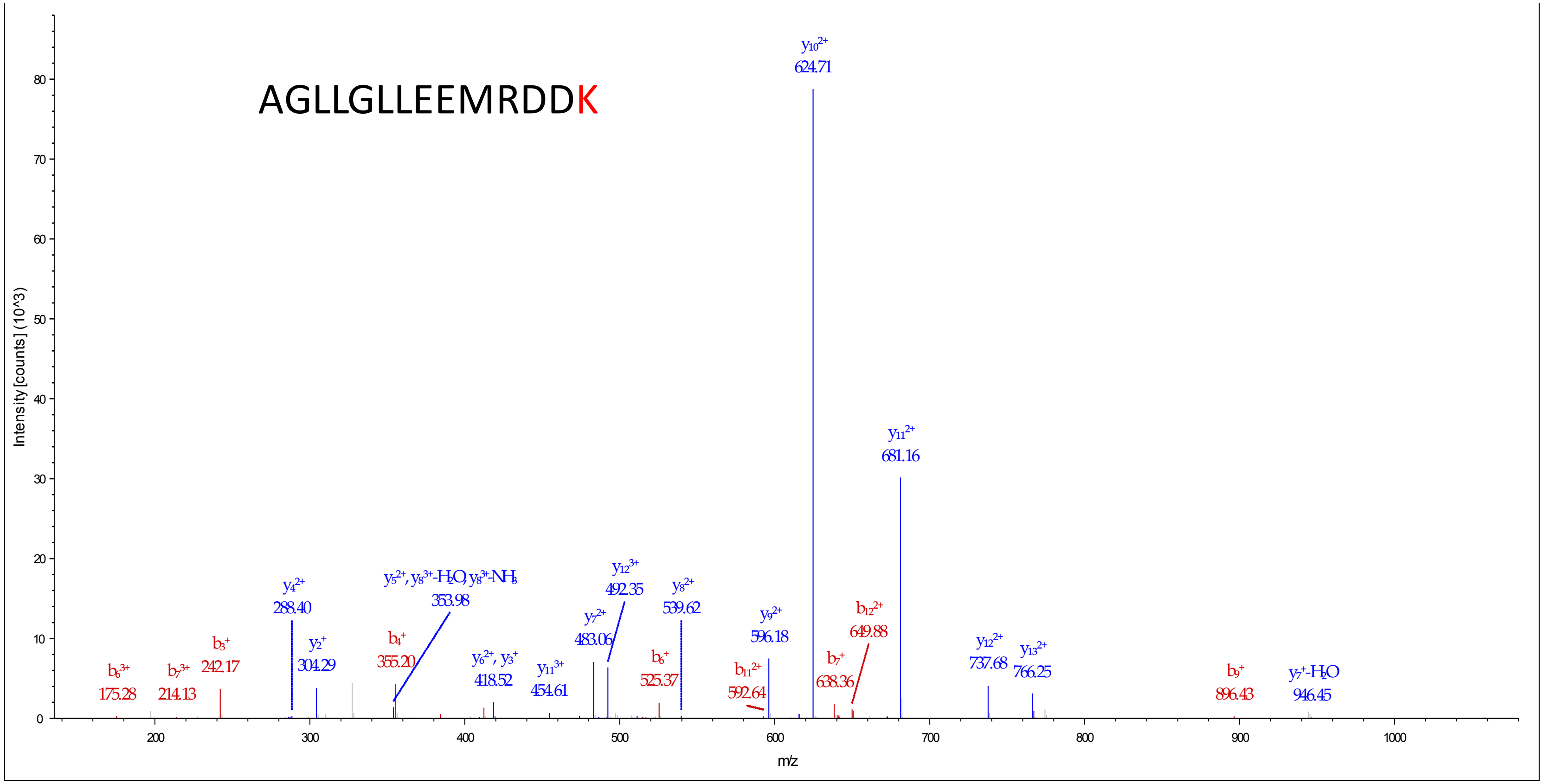

(22)

Figure S1. *Cont.*

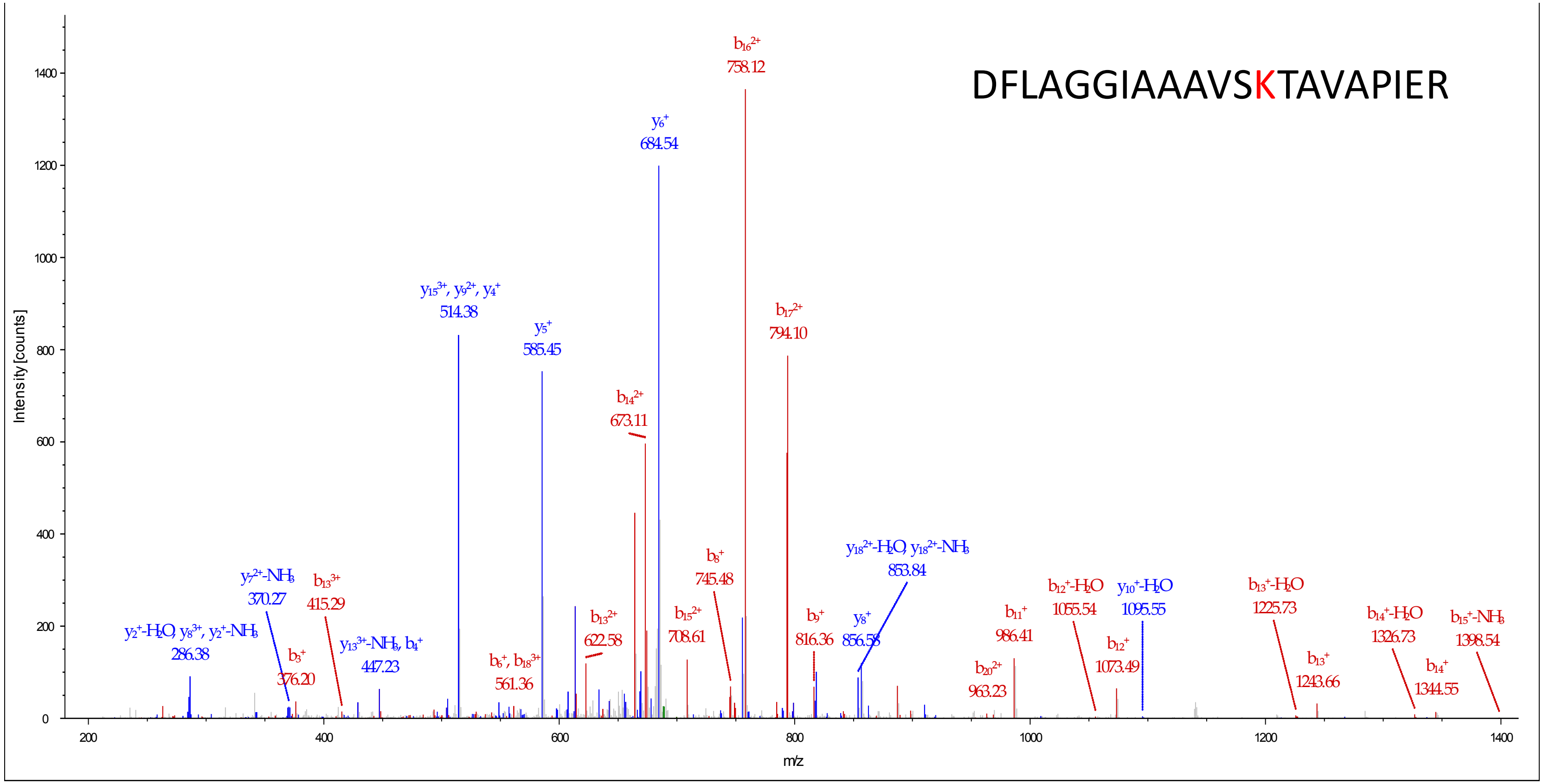

(23)

Figure S1. *Cont.*

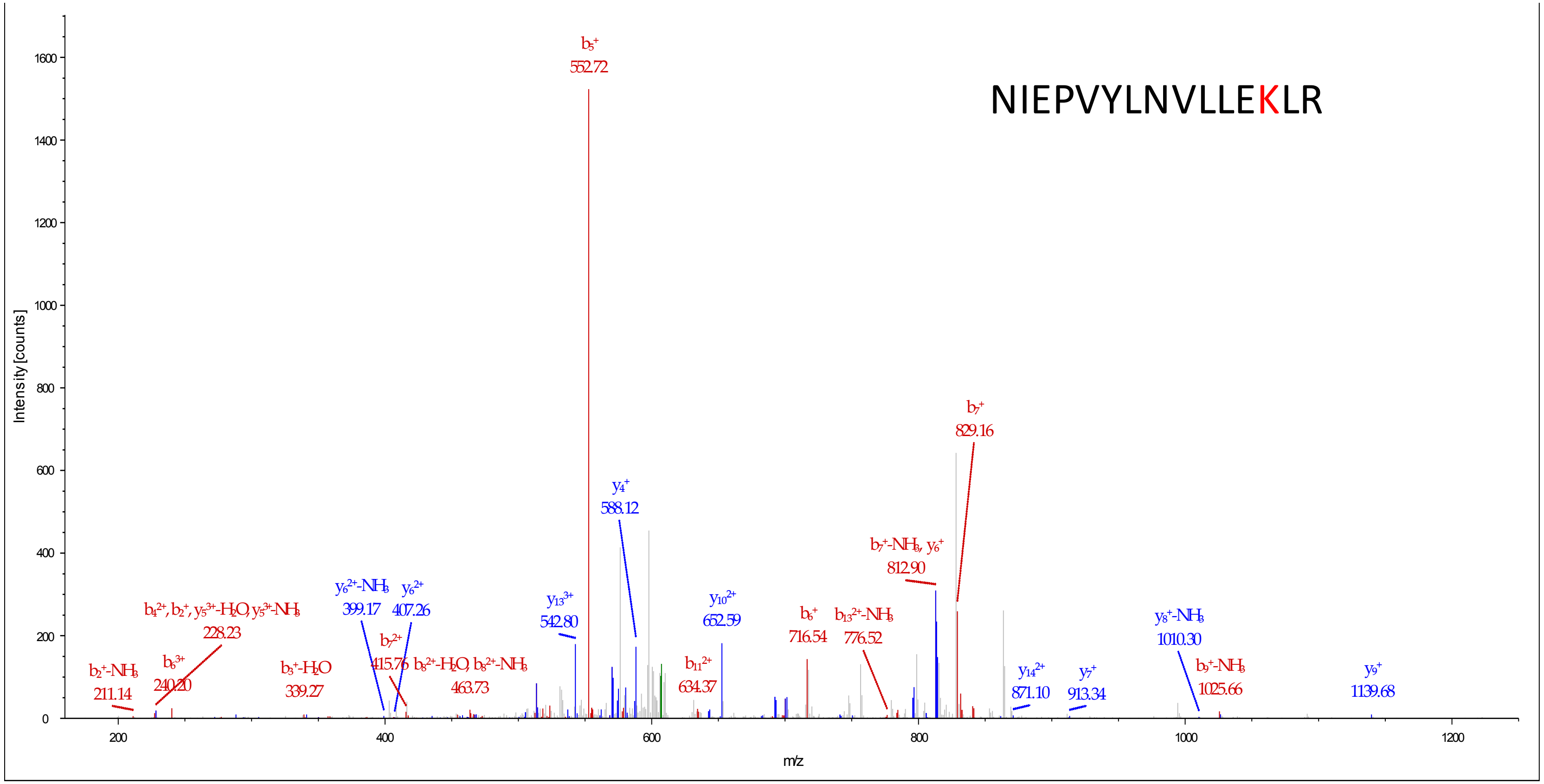

(24)

Figure S1. *Cont.*

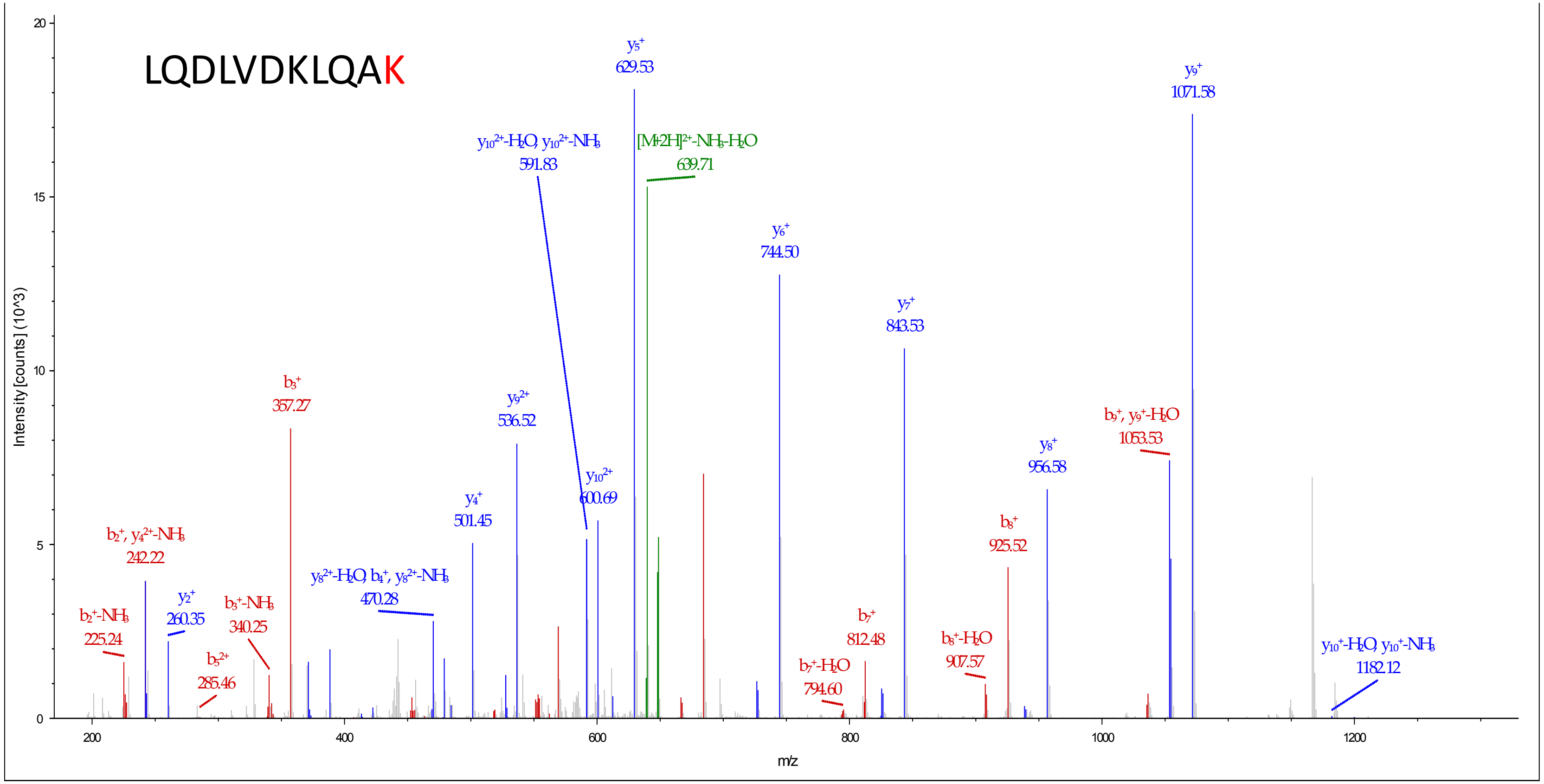

(25)

Figure S1. *Cont.*

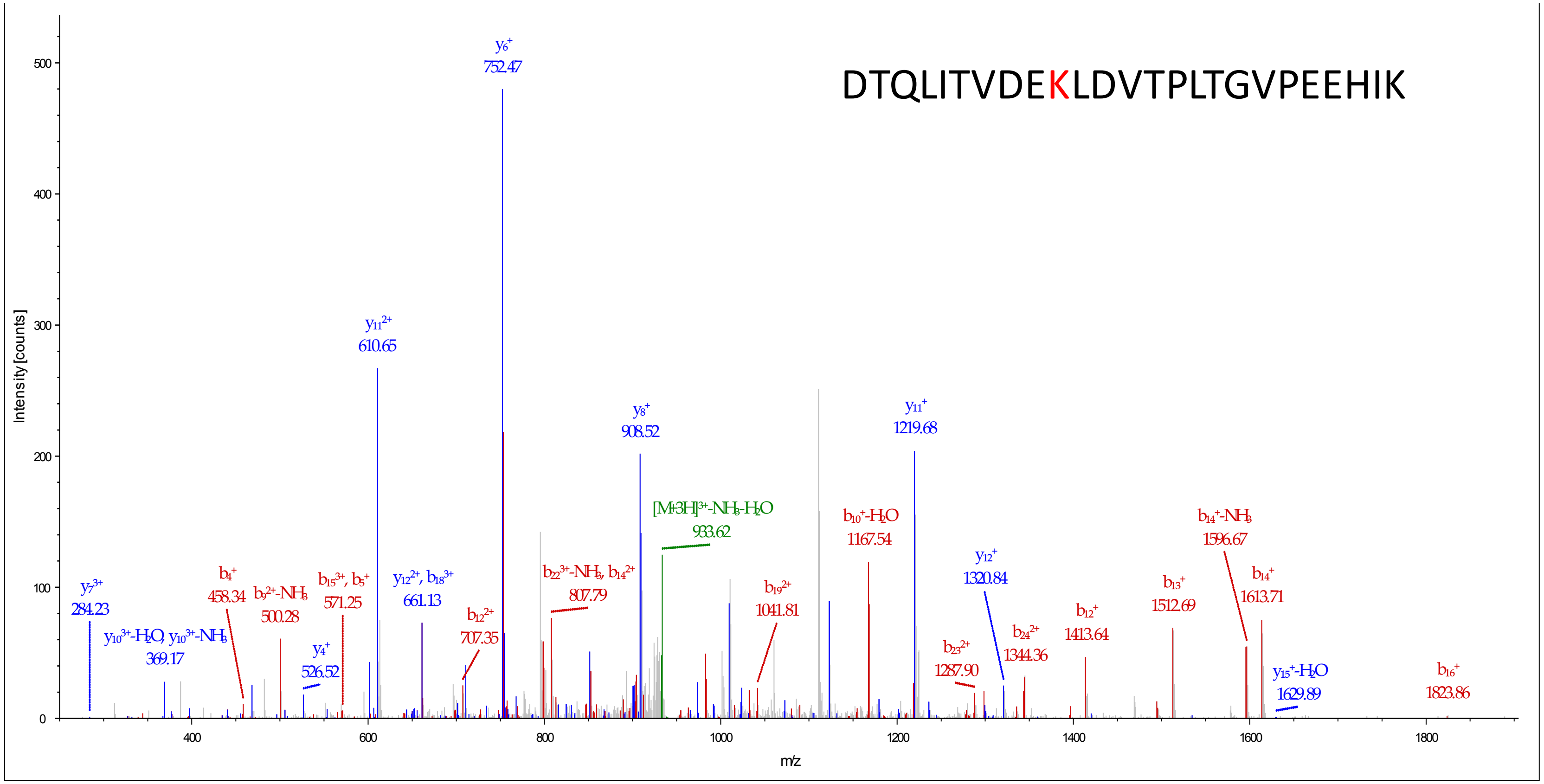

(26)

Figure S1. *Cont.*

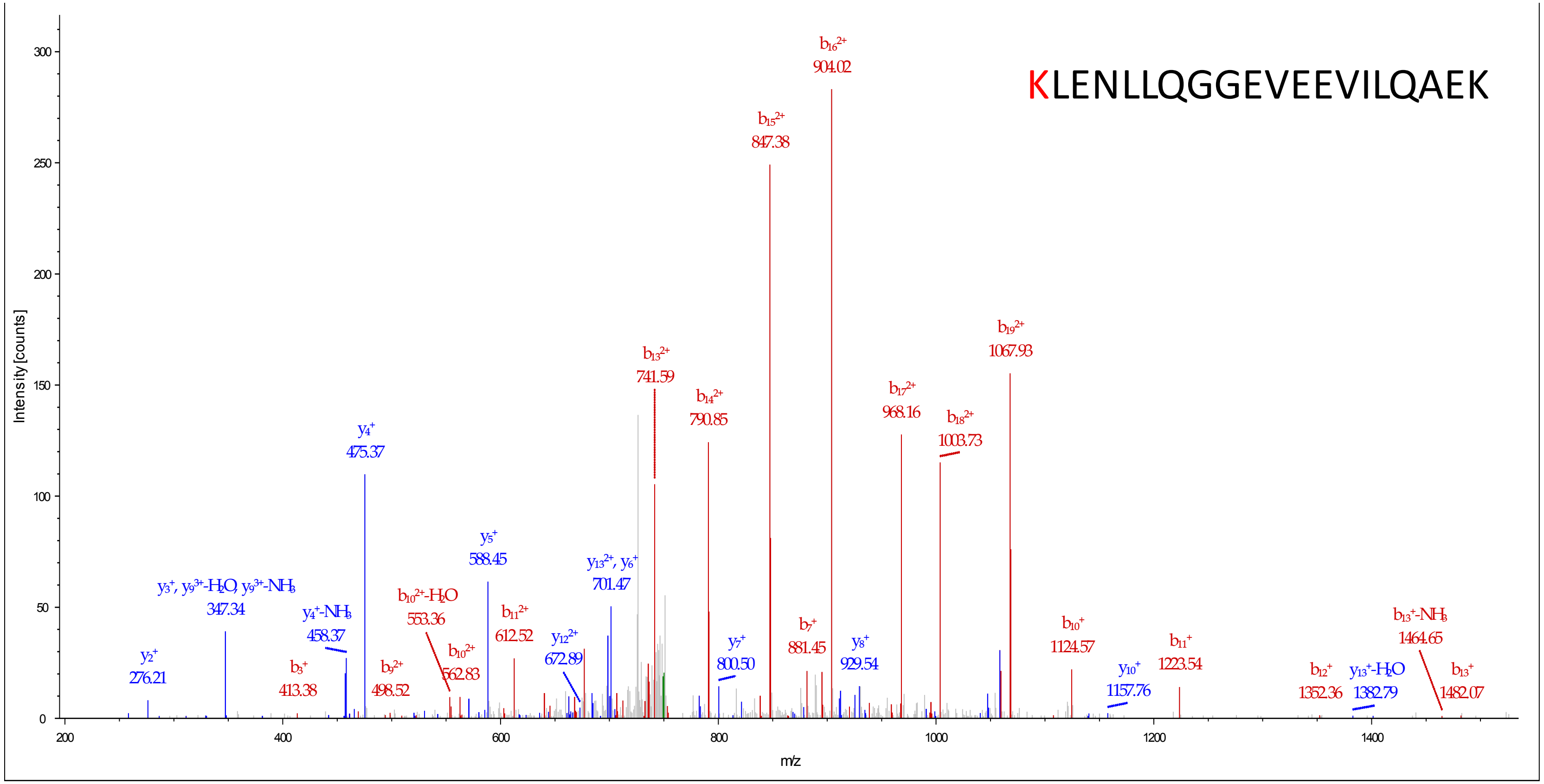

(27)

Figure S1. *Cont.*

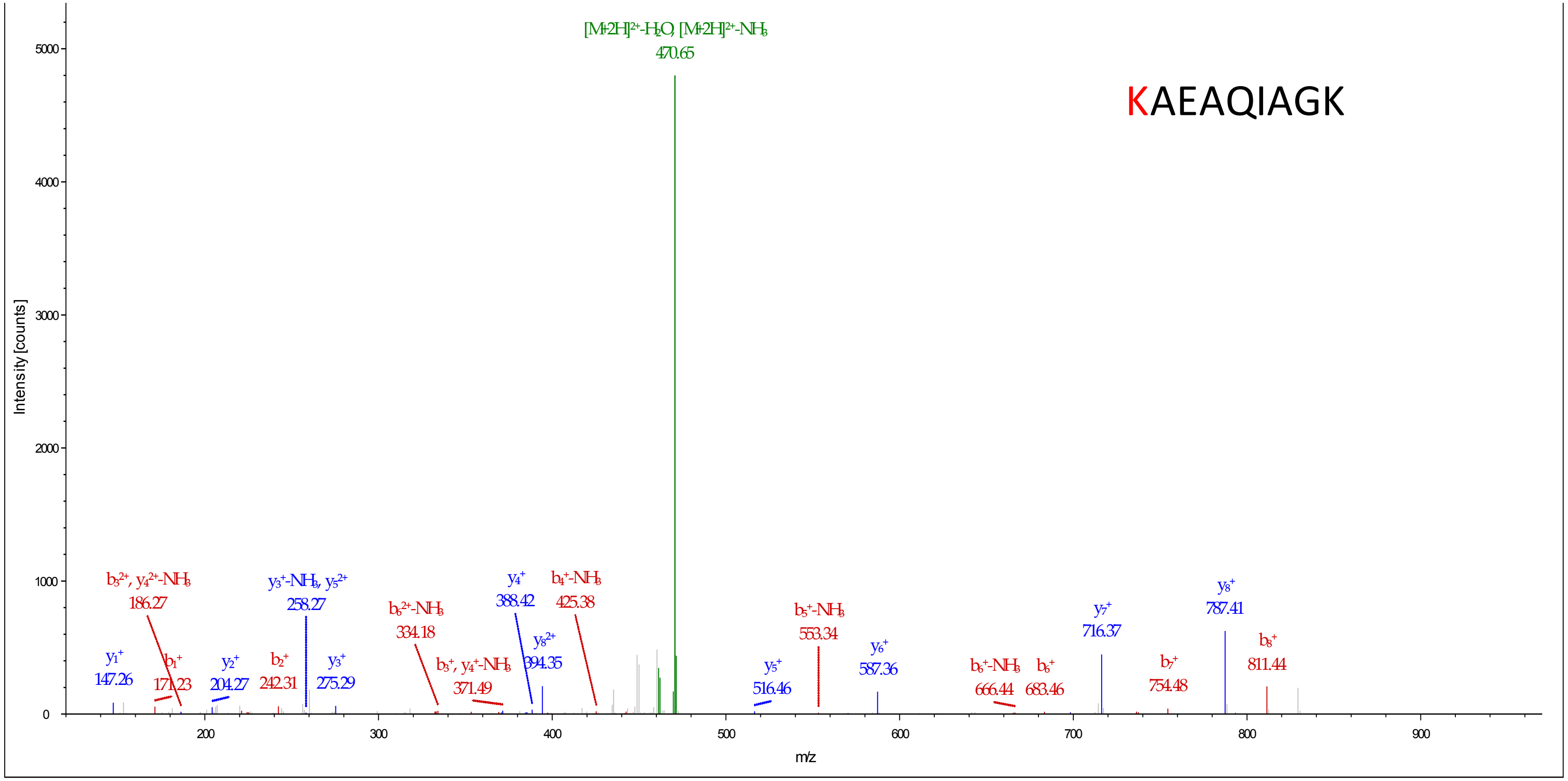

(28)

Figure S1. *Cont.*

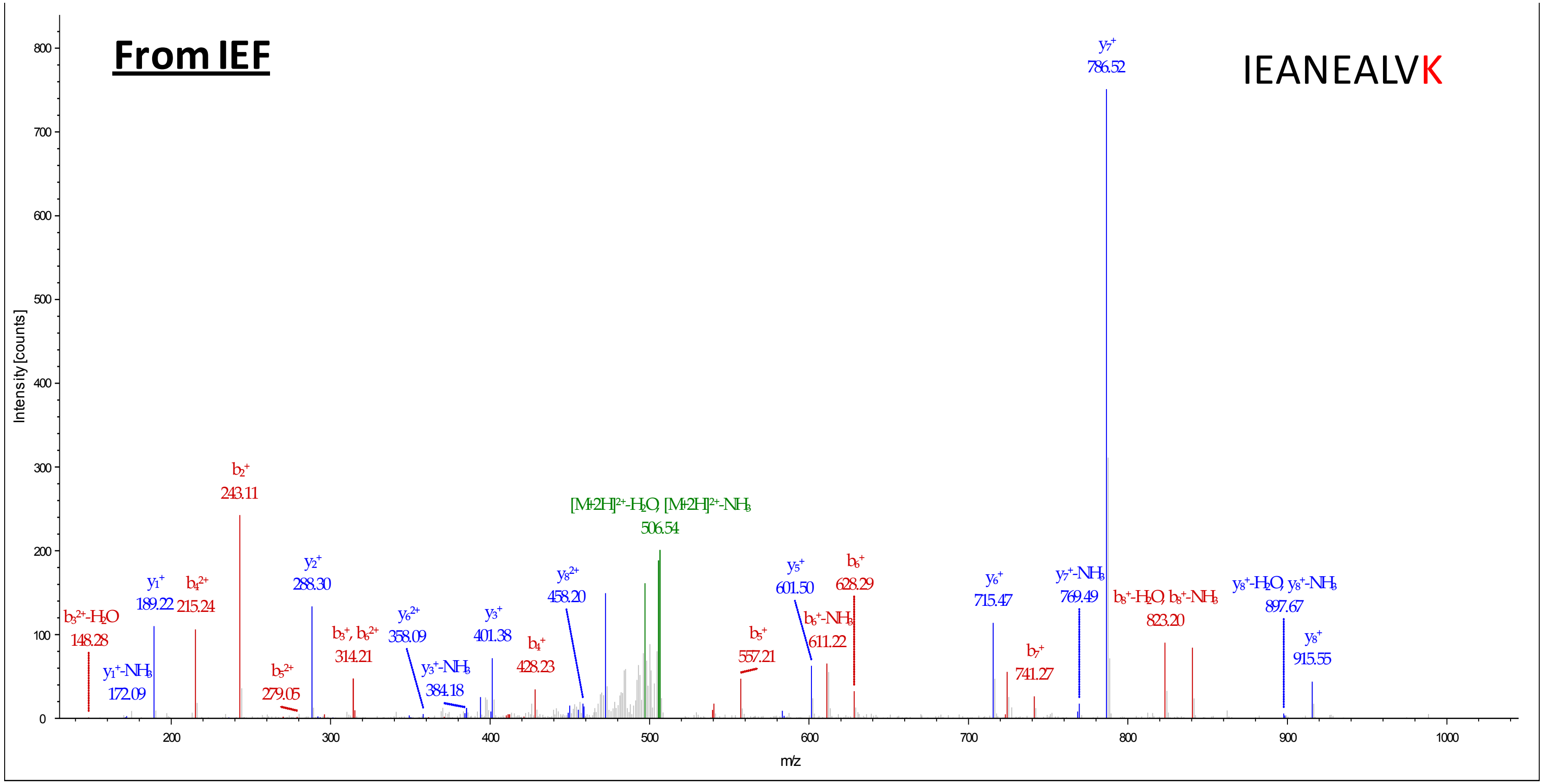

(**29**)

Figure S1. *Cont.*

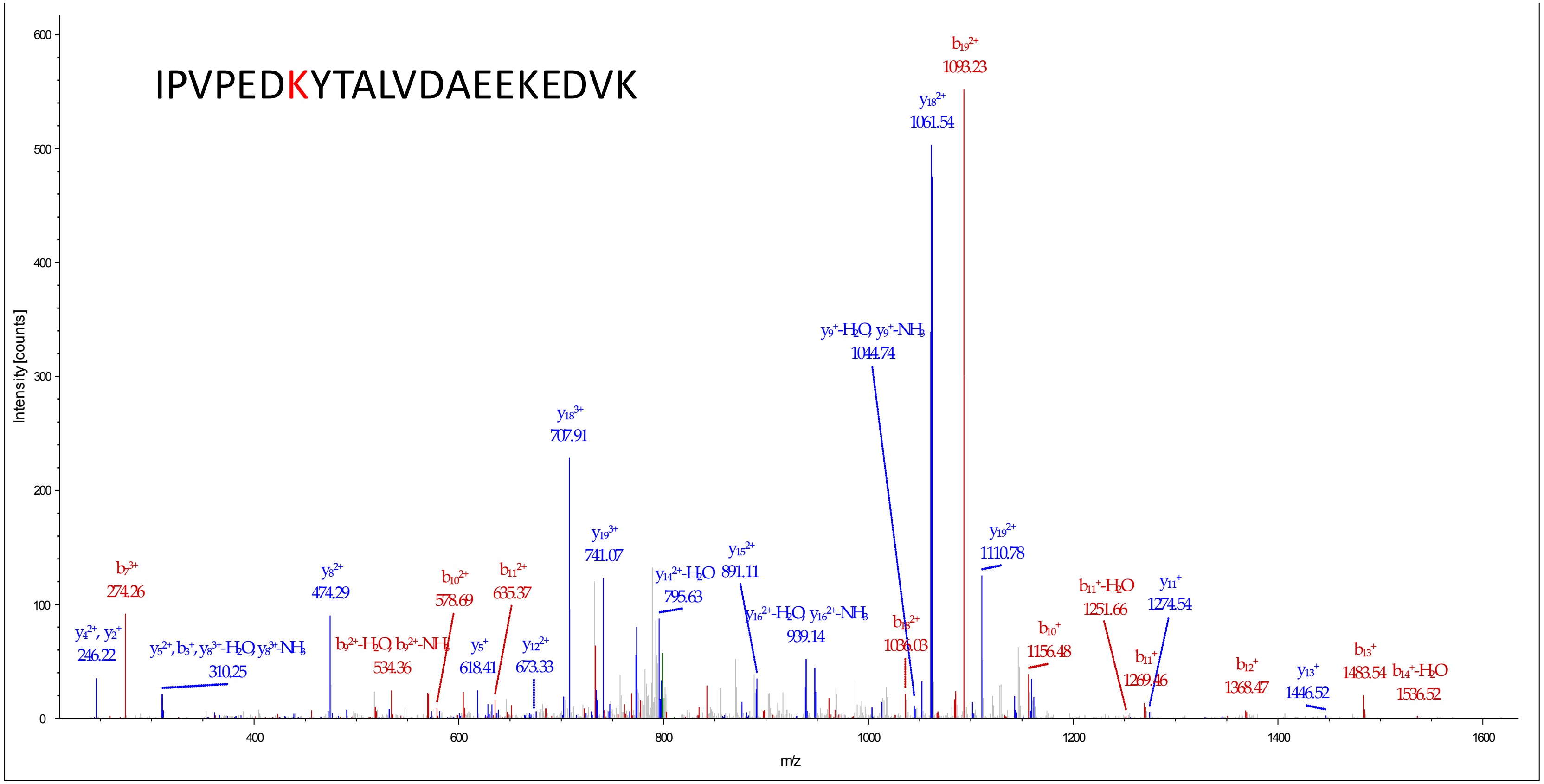

(30)

Figure S1. *Cont.*

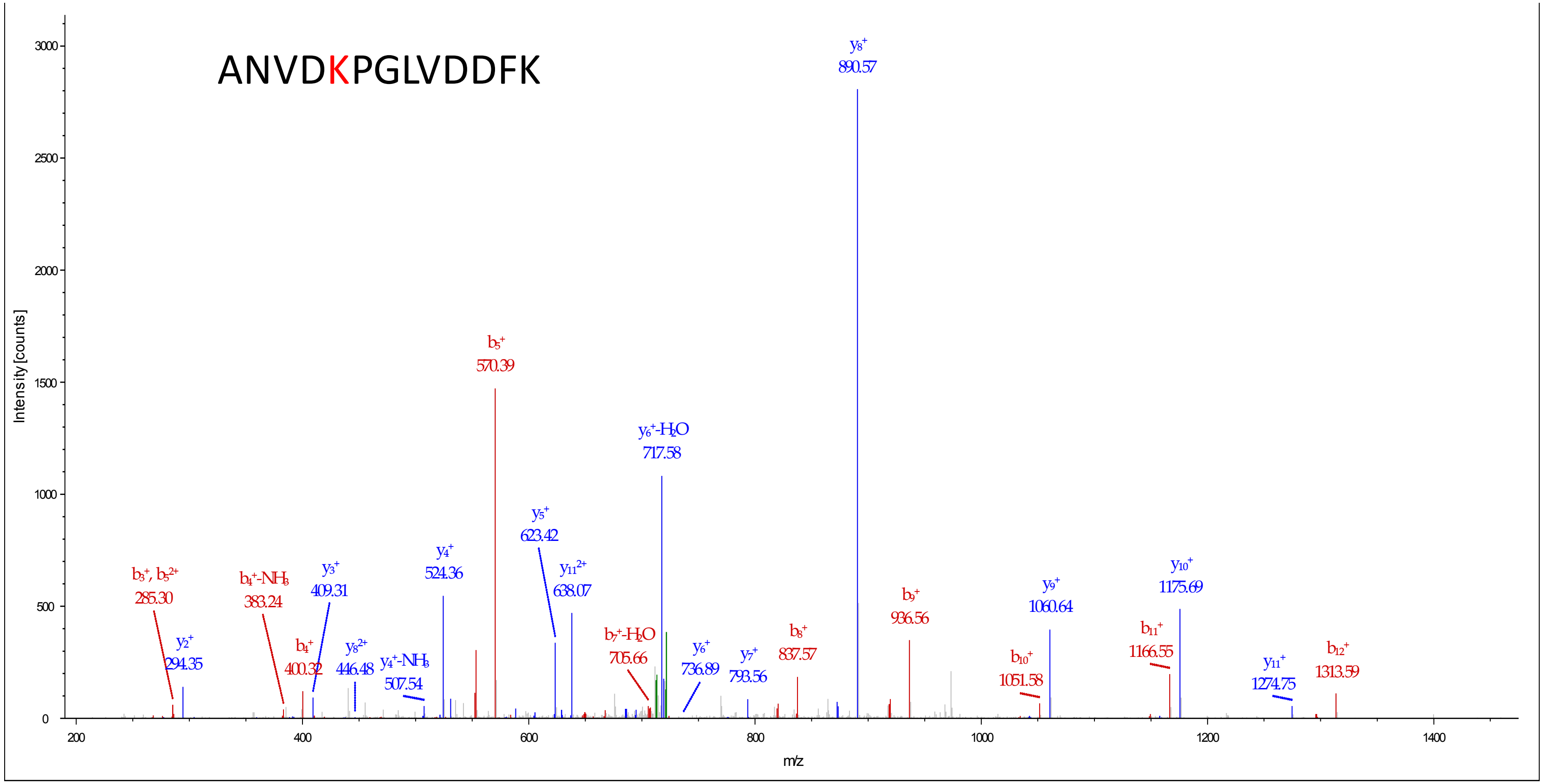

(31)

Figure S1. *Cont.*

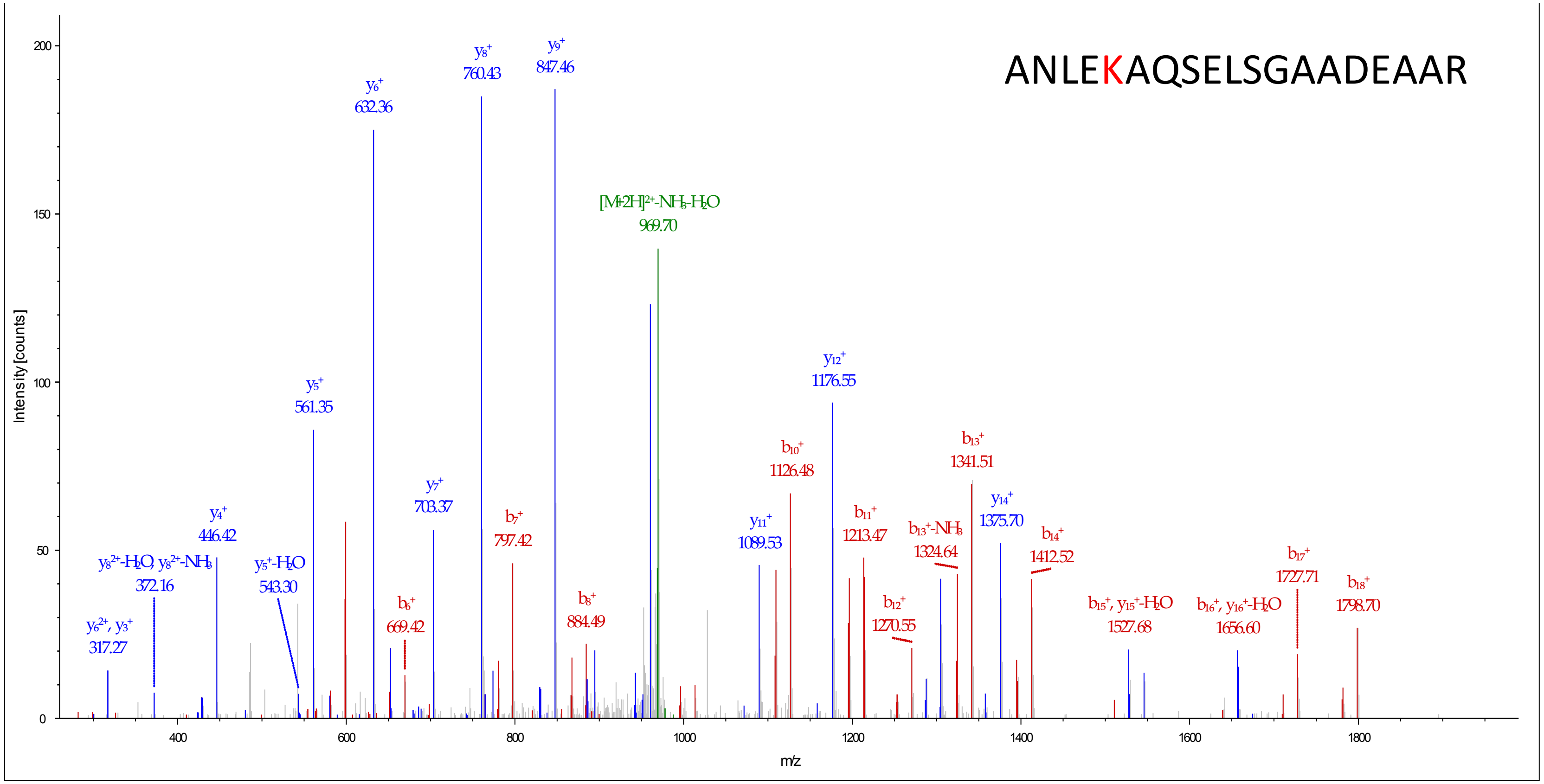

(32)

Figure S1. *Cont.*

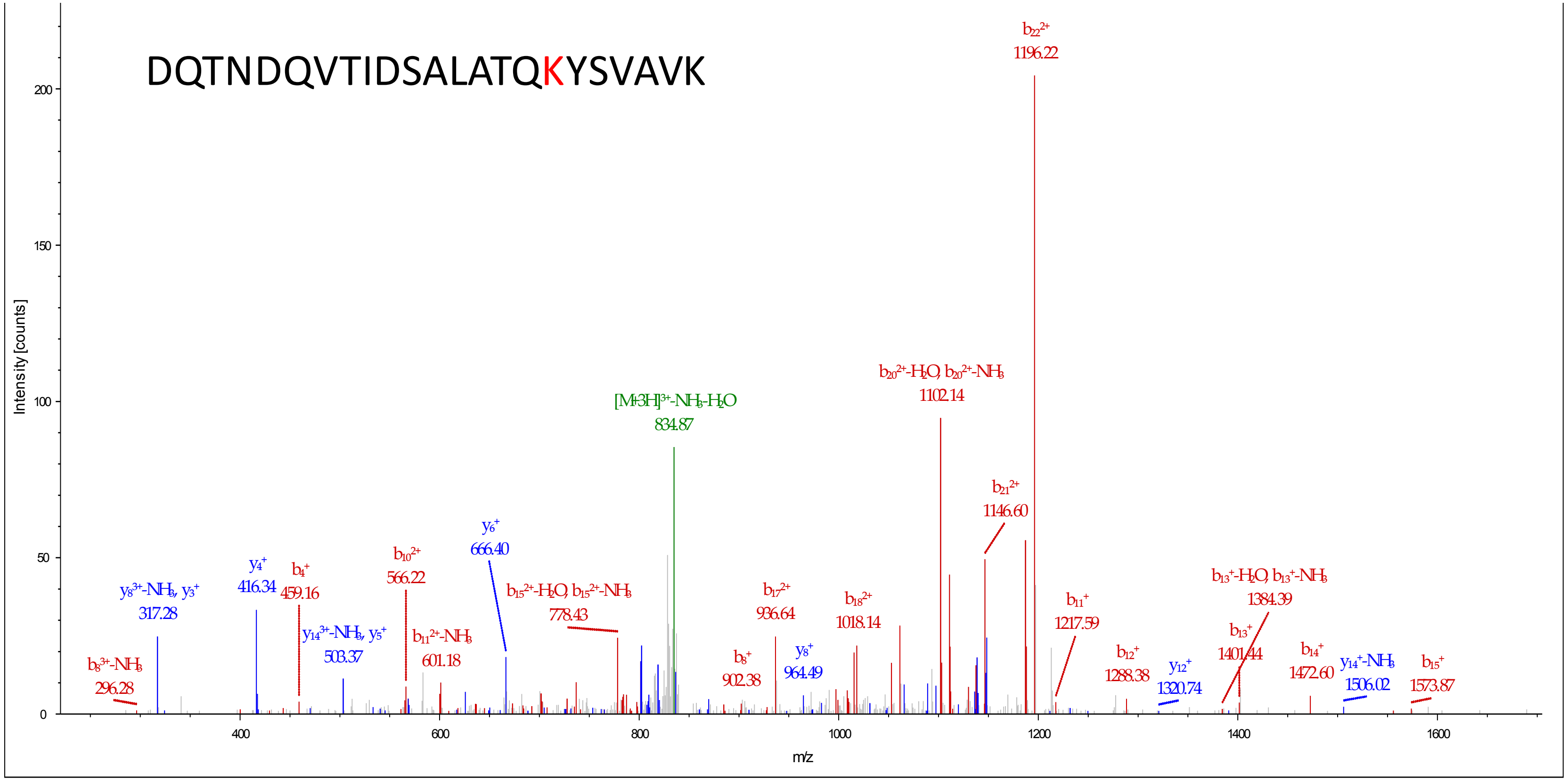

(33)

Figure S1. *Cont.*

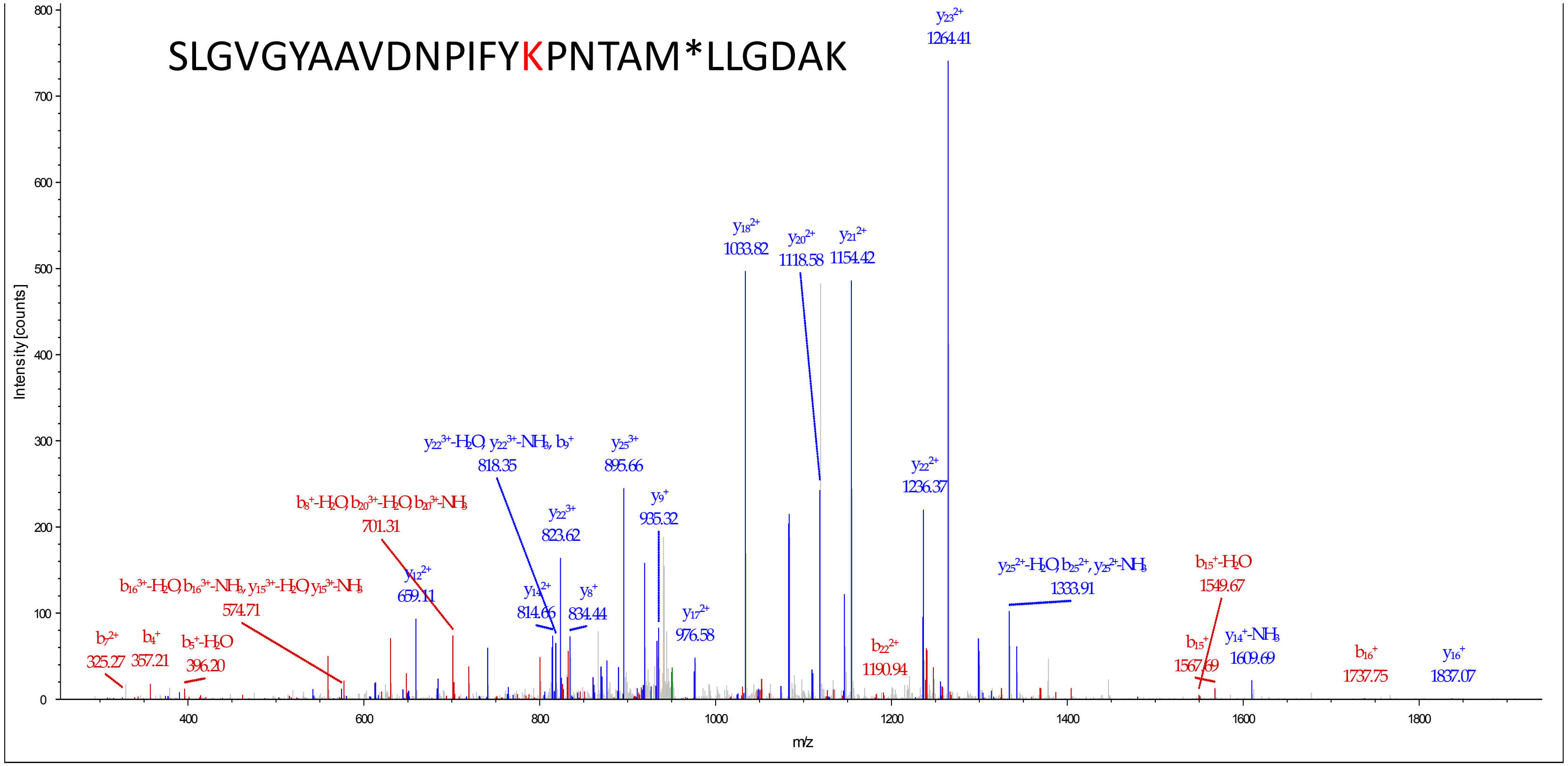

(34)

Figure S1. *Cont.*

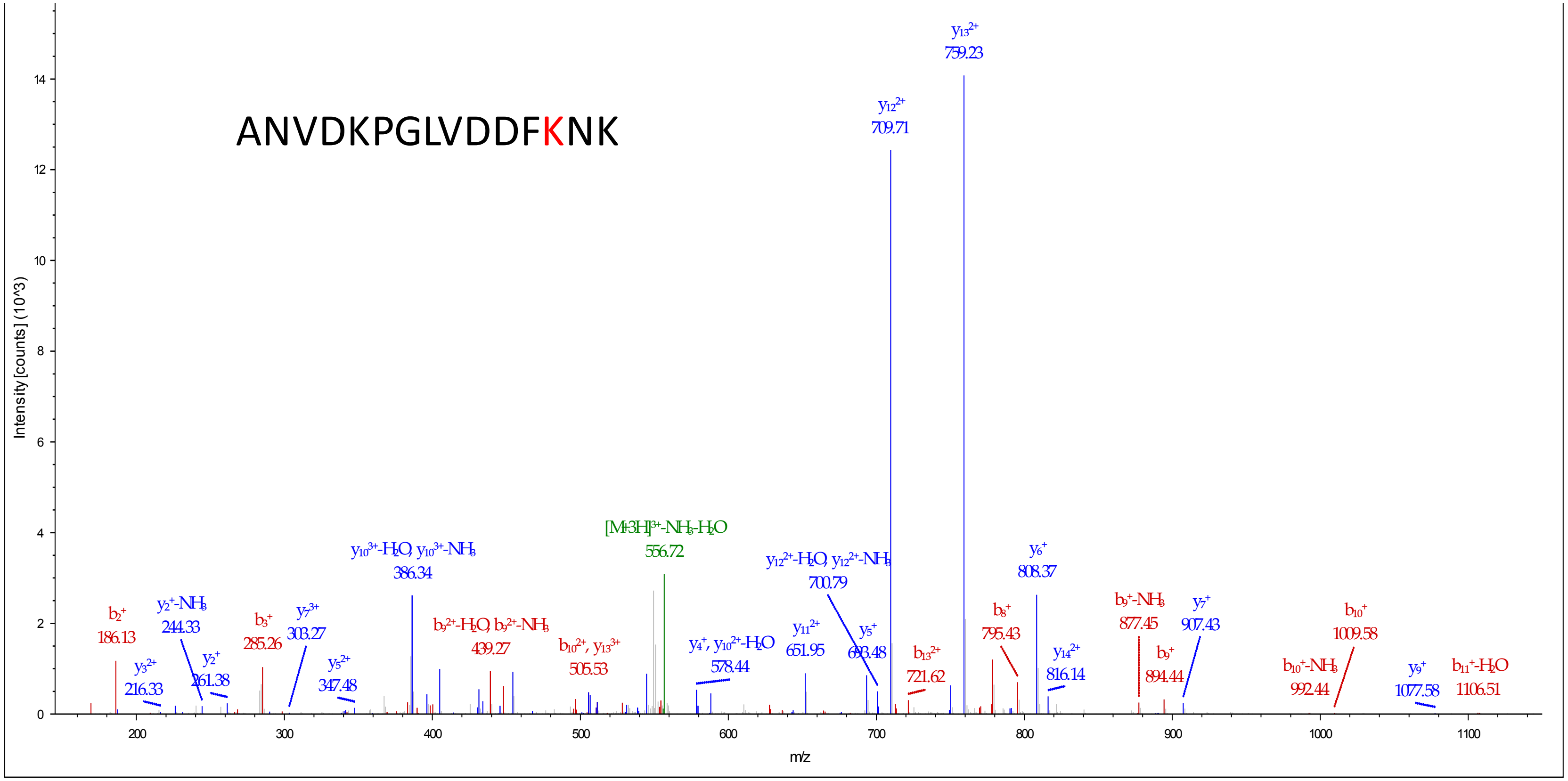

(35)

Figure S1. *Cont.*

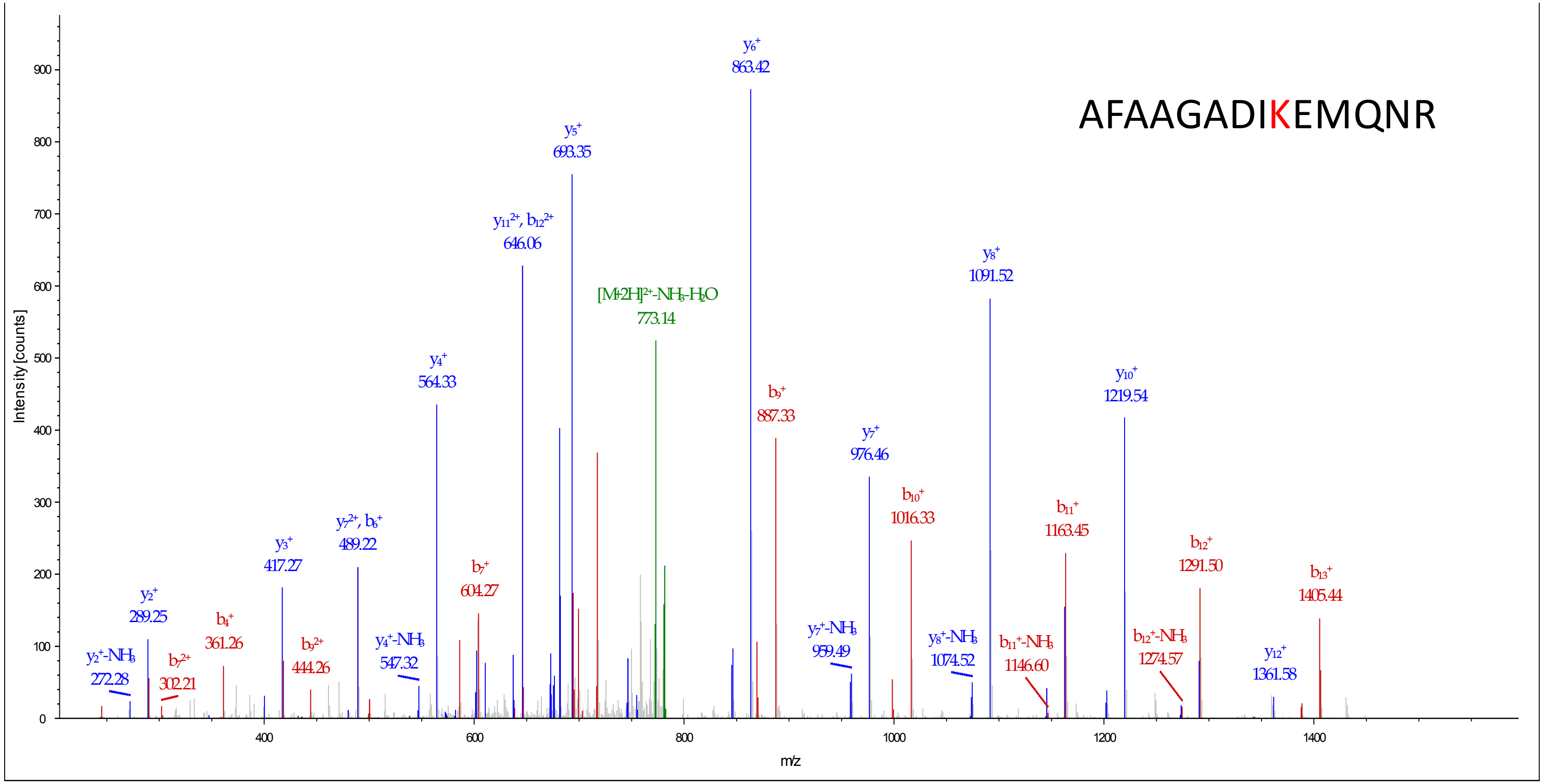

(36)

Figure S1. *Cont.*

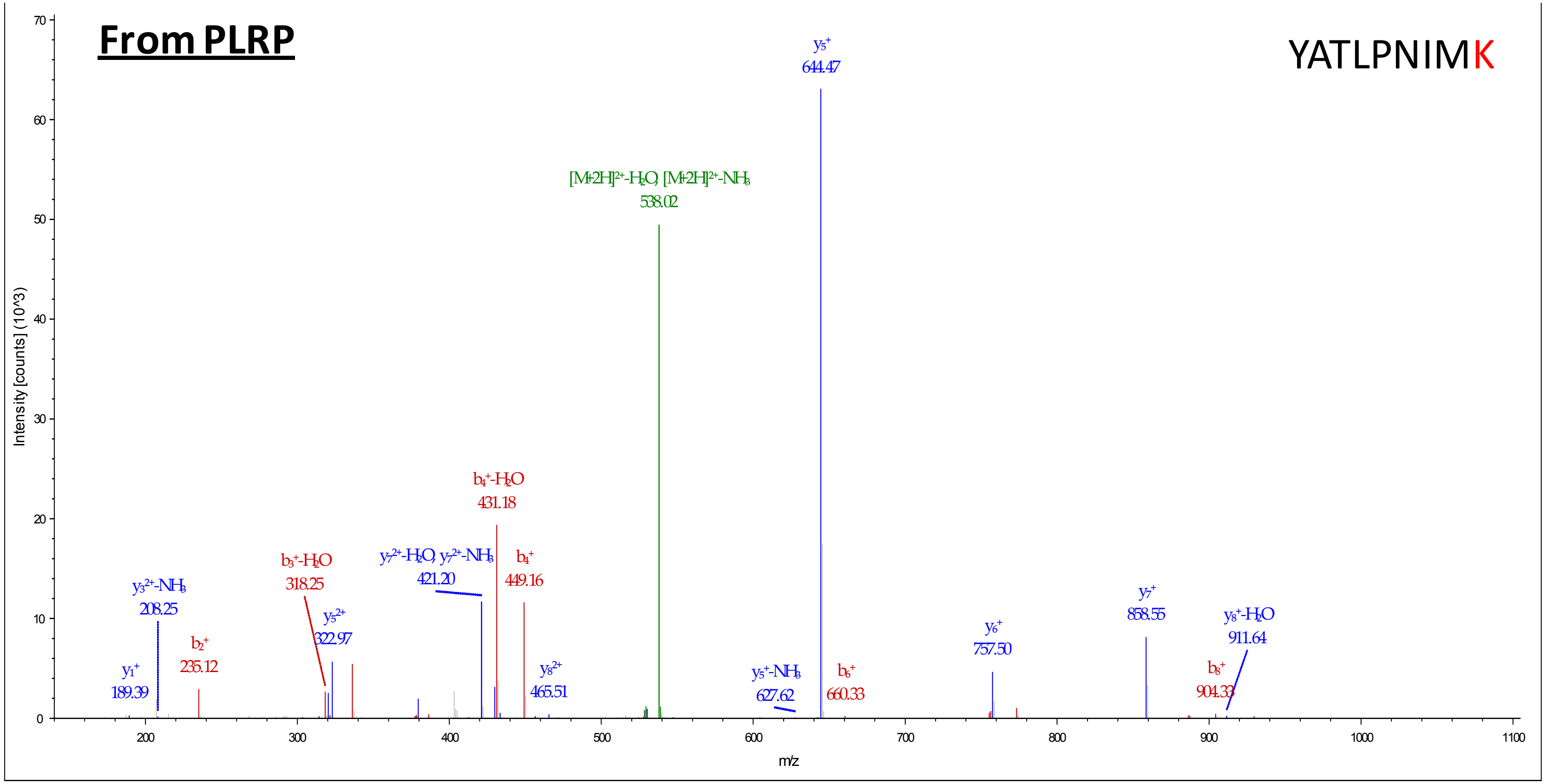

(37)

Figure S1. *Cont.*

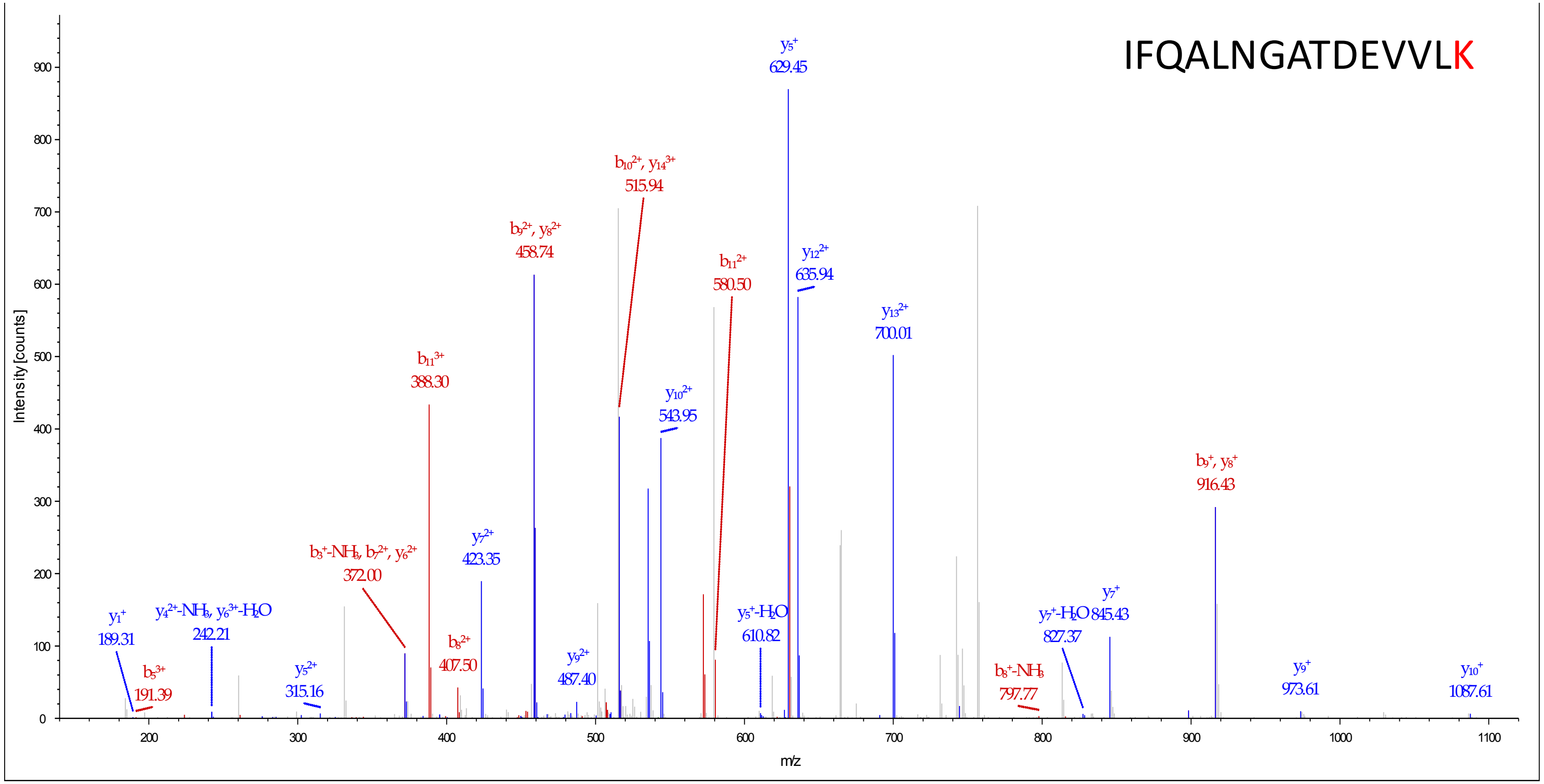

(38)

Figure S1. *Cont.*

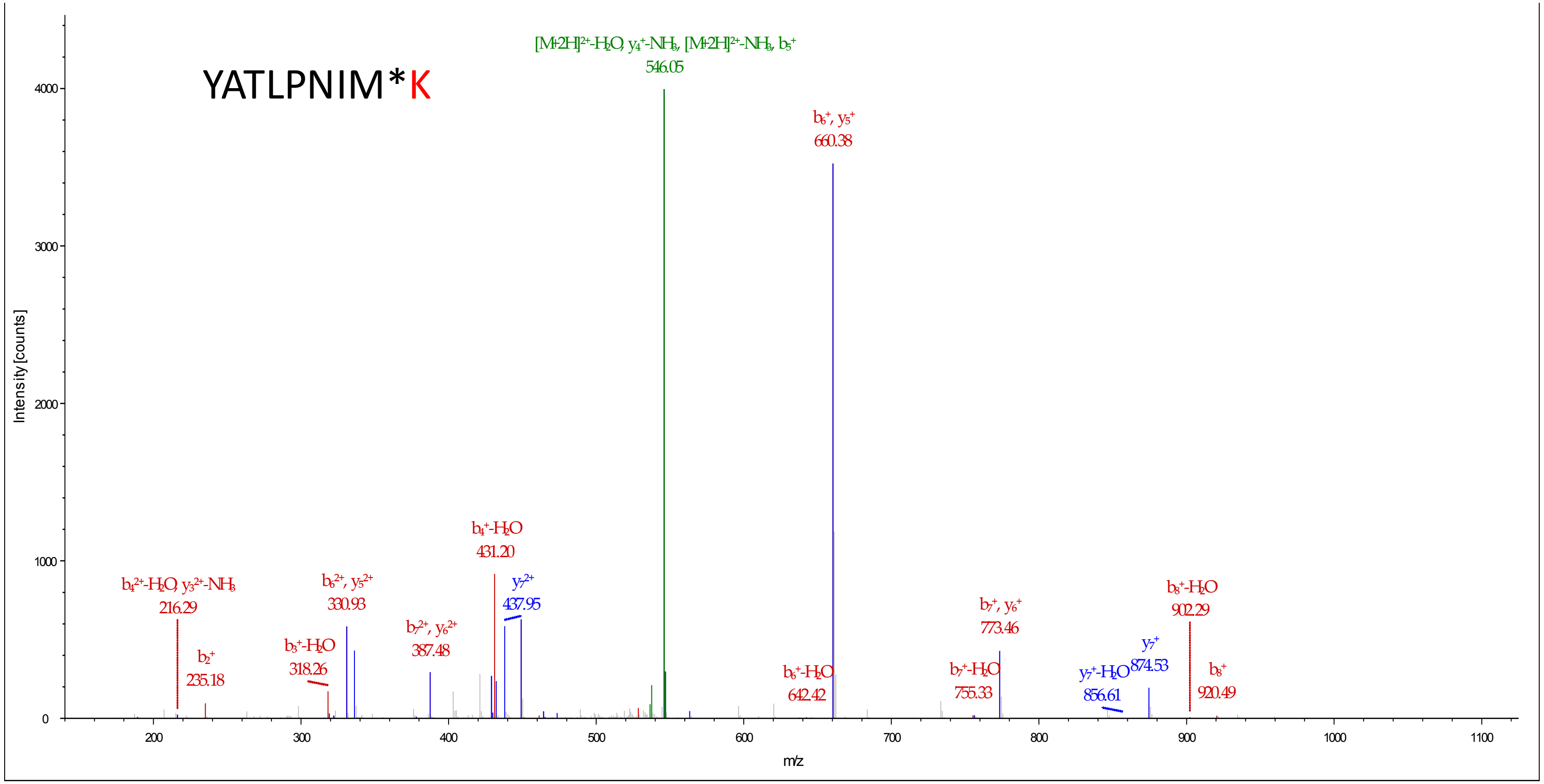

(39)

© 2014 by the authors; licensee MDPI, Basel, Switzerland. This article is an open access article distributed under the terms and conditions of the Creative Commons Attribution license (http://creativecommons.org/licenses/by/3.0/).
